# Quantifying Shared and Unique Gene Content across 17 Microbial Ecosystems

**DOI:** 10.1128/msystems.00118-23

**Published:** 2023-04-06

**Authors:** Samuel Zimmerman, Braden T. Tierney, Chirag J. Patel, Aleksandar D. Kostic

**Affiliations:** a Section on Pathophysiology and Molecular Pharmacology, Joslin Diabetes Center, Boston, Massachusetts, USA; b Section on Islet Cell and Regenerative Biology, Joslin Diabetes Center, Boston, Massachusetts, USA; c Department of Microbiology, Harvard Medical School, Boston, Massachusetts, USA; d Department of Biomedical Informatics, Harvard Medical School, Boston, Massachusetts, USA; NYU Langone Health

**Keywords:** bioinformatics, human microbiome, metagenomics

## Abstract

Measuring microbial diversity is traditionally based on microbe taxonomy. Here, in contrast, we aimed to quantify heterogeneity in microbial gene content across 14,183 metagenomic samples spanning 17 ecologies, including 6 human associated, 7 nonhuman host associated, and 4 in other nonhuman host environments. In total, we identified 117,629,181 nonredundant genes. The vast majority of genes (66%) occurred in only one sample (i.e., “singletons”). In contrast, we found 1,864 sequences present in every metagenome, but not necessarily every bacterial genome. Additionally, we report data sets of other ecology-associated genes (e.g., abundant in only gut ecosystems) and simultaneously demonstrated that prior microbiome gene catalogs are both incomplete and inaccurately cluster microbial genetic life (e.g., at gene sequence identities that are too restrictive). We provide our results and the sets of environmentally differentiating genes described above at http://www.microbial-genes.bio.

**IMPORTANCE** The amount of shared genetic elements has not been quantified between the human microbiome and other host- and non-host-associated microbiomes. Here, we made a gene catalog of 17 different microbial ecosystems and compared them. We show that most species shared between environment and human gut microbiomes are pathogens and that prior gene catalogs described as “nearly complete” are far from it. Additionally, over two-thirds of all genes only appear in a single sample, and only 1,864 genes (0.001%) are found in all types of metagenomes. These results highlight the large diversity between metagenomes and reveal a new, rare class of genes, those found in every type of metagenome, but not every microbial genome.

## INTRODUCTION

The human microbiome, with its established role in modulating host health (1–4), is diverse and arguably a multitude of different ecosystems. Moreover, the microorganisms that inhabit these niches are in no way limited to a single locale across space and time. Many microbes are transient (5, 6), passing through the gut, for example, and others, even if colonizing long term, evolved from ancestors that lived in other hosts or environments altogether (7). The taxonomic, functional, and genetic content of the human microbiome must be contextualized across ecologies to estimate evolutionary history relative to the universe of microbiological life. Public databases that include more than one body site or host are critical in understanding the ecology of microbiomes *writ large*.

These resources enable answers to basic questions about microbes and their role in human health. Comparisons between human microbiome content, most frequently the human gut, and environmental metagenomes have yielded important findings for microbiome ecology in the past, both in terms of the overall taxonomic similarity of ecosystems (at the level of the *16S* gene) as well as the development of its role in host health (8). For example, to the latter point, one group used a combination of microbiome data sources to track the evolutionary history of *Enterococcus* across time, identifying how it adapted to become virulent (9). Other teams have purported the “hygiene hypothesis,” which argues that exposure to certain microbes at a young age can be protective against autoimmune diseases (10). However, testing the hygiene hypothesis requires a thorough understanding of the interactions between environmental and human microbial ecologies.

There is a need to understand the diversity and distribution of bacterial proteins—as opposed to taxonomies—across metagenomic life. In this report, we refer to open reading frames (ORFs) grouped into clusters by sequence identity and represented by a consensus sequence as “genes.” Some public databases (e.g., UniProt) (11, 12) contain genes measured across ecologies and environments; however, these databases are not designed to address higher-resolution comparative biological questions, including enrichment or conservation of specific sequences or functions across different environments. Many other published gene databases focus on single niches, predominantly gut ecosystems (13).

Additional similar resources tend to focus on taxonomies or genome reconstruction, such as those comprising metagenome-assembled genomes (MAGs) (14–16). Compared to MAGs, databases of pan-ecological microbial gene content are lacking. Publications focusing on MAGs have provided insight into the genomic structure of gene content of metagenomes across environments, but by virtue of building a MAG, one loses potentially interesting genetic information in genes that are not placeable within a genome. For example, MAGs have been hypothesized to exclude certain bacterial genes (17). While there are recent impressive databases that compare across environments, few, if any, have attempted to explicitly report the “core” genes enriched in different ecosystems (or found in all of them) (16).

Genes, after all, (i) underpin the functional processes of a microbe, (ii) encode specific protein products (e.g., antibiotics and other small molecules) of biotechnological importance (18, 19), and (iii) have been shown, in our prior work, to outperform taxonomy-based metrics for predicting host phenotypes (4, 20). Finally, a gene-centric view of microbial ecosystems provides one agnostic to the much-debated “microbial species concept” and sidesteps the difficult challenge of quantifying genome “completeness” (21).

Contextualizing the gene universe of metagenomics across ecologies allows one to look into the “conserved” structure of microbial life in terms of genes that are shared between some or all metagenomic ecosystems. Prior work has revealed sets of conserved genes across bacteria (22) and all three domains of life with various levels of success (23, 24). However, these studies search for conserved genes in a relatively small set of evolutionarily distant organisms. To our knowledge, a search for universally conserved proteins across thousands of metagenomes in different ecologies has not been executed. These hypothetical genes, however, could serve an important purpose through, for example, the “black queen” hypothesis, where functions essential to a community are found not in every cell, but an isolated number of organisms (25).

Here, we explore the gene universe of metagenomic data, with access to metadata (e.g., host disease state), various analytic strategies (e.g., genes clustered at different percent identities), and data sets of ecology-specific genes. Specifically, we aimed to quantify the global gene-level (as opposed to taxonomic) similarity between human, other host-associated, and environmental metagenomes, with a focus on (i) measuring singleton content across environments, (ii) characterizing and building databases of genes enriched in different metagenome types, and (iii) estimating the number of microbial genes that are conserved across every metagenome, but not every microbial genome. We additionally ran our analysis at various clustering parameters in order to identify the “optimal” clustering identity for gene catalogs.

## RESULTS

### A pan-ecological, gene-centric database of metagenomic life.

**FIG 1:**
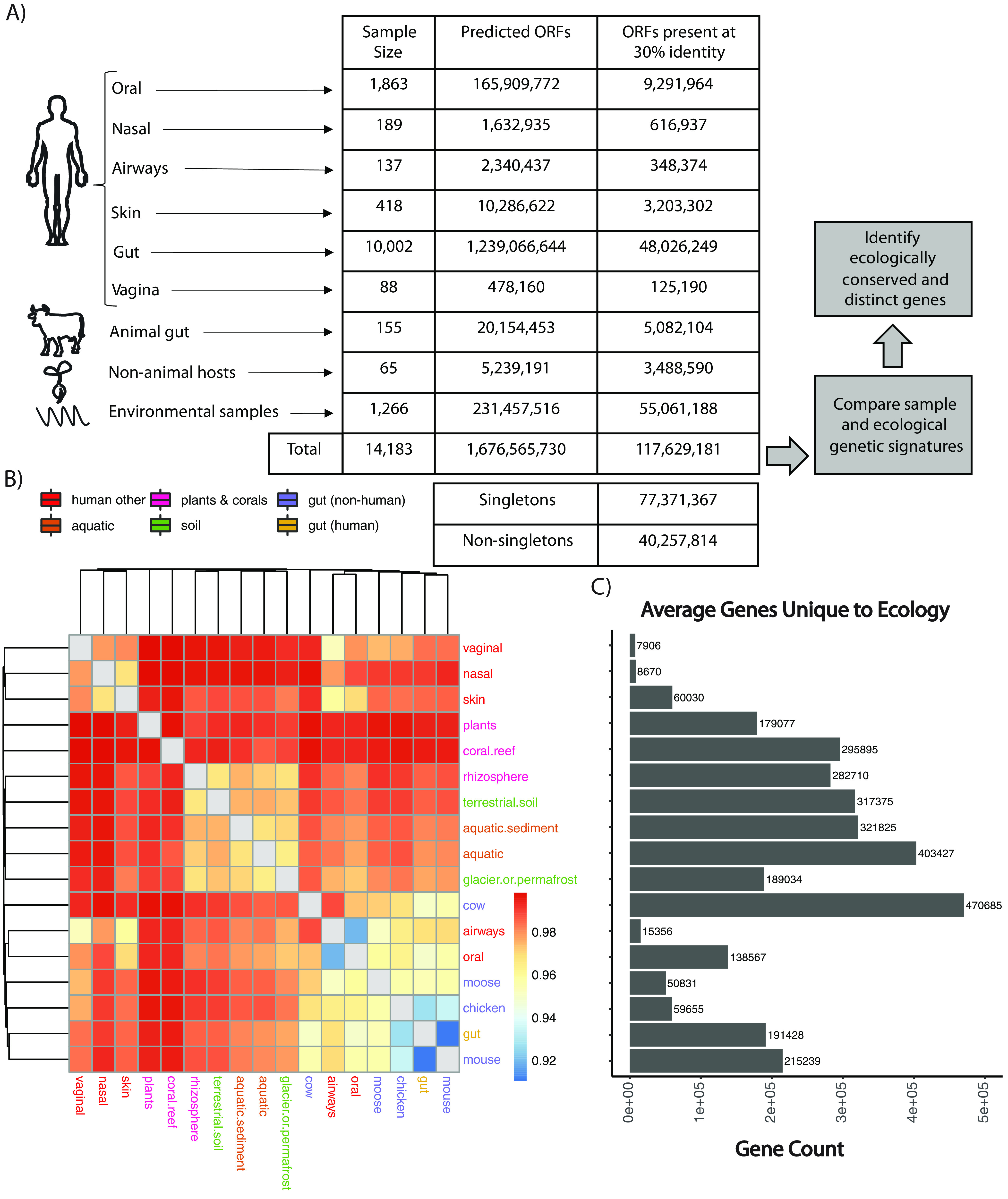
Overview of approach and results and genetic similarity between ecologies. (A) Statistics regarding the gene and sample content of our database at the 30% clustered sequence identity and the high-level analytical steps we took in the manuscript. (B) Hierarchical clustering on the Jaccard distance between ecologies as a function of iterative sampling. Each cell represents the average Jaccard distance between two ecologies (in rows and columns) after 50 random samplings. Cell color is in units of Jaccard distance. The color of text corresponds to broader ecology class. (C) Average number of genes unique to (found only in) a given ecology.

We predicted microbial genes across 17 different ecologies from 14,183 samples (see Table S1 in the supplemental material). We used publicly available metagenomic samples and assemblies, identifying 1,676,565,730 raw ORFs from assembled contigs. We built a series of nonredundant (nr) gene catalogs at various amino acid identities (from 100% to 30%). At the 30% amino acid identity (see Materials and Methods), we identified 117,629,181 nonredundant genes (Fig. 1A), which had a total of 15,712 unique protein annotations, 2,628 unique Cluster of Ortholog Genes (COG) annotations, and 2,820 unique EC numbers. Sixty-one percent of these genes were novel, as they were not found in other databases used for gene annotation (Fig. S1B). We quantified the number of nonredundant genes found in each ecology, noting that larger sample sizes, as expected, yielded an increase in the number of genes identified. At 50% identity and 90% identity, we identified a total of 147,534,185 and 254,624,293 nonredundant genes, respectively.

10.1128/msystems.00118-23.1 FIG S1 Percentage of novel genes in the gene catalog. (A) We calculated the proportion of genes in other common gene catalogs (Coelho et al. [46], OrthoDB, and KEGG) that were in our 30% gene catalog, as well as the proportion of genes in our 30% gene catalog that were in other common gene catalogs such as Coelho et al. (46), OrthoDB, KEGG, and eggNog (B). Download FIG S1, PDF file, 0.4 MB. Copyright © 2023 Zimmerman et al. 2023 Zimmerman et al. https://creativecommons.org/licenses/by/4.0/ This content is distributed under the terms of the Creative Commons Attribution 4.0 International license.

10.1128/msystems.00118-23.7 TABLE S1 Sample metadata and summary statistics. File containing database summary information (e.g., *N*_50_s, sample IDs, study IDs, number of genes per sample) as well as sample metadata (e.g., disease or Westernization status). Download Table S1, XLSX file, 3.5 MB. Copyright © 2023 Zimmerman et al. 2023 Zimmerman et al. https://creativecommons.org/licenses/by/4.0/ This content is distributed under the terms of the Creative Commons Attribution 4.0 International license.

All nonredundant gene catalogs are hosted at http://www.microbial-genes.bio. Further, in an effort to provide “environmentally contextualized” data for different genes and metagenomes, we have made available specific gene sets of interest highlighted by the analysis that follows along with host sample characteristics (e.g., host subject age, sex, and disease status). We annotate each gene ID, for example, being conserved across all ecologies or unique to specific ecological subsets.

We counted the proportion of “singleton” genes (26). Even with a lower percent identity cutoff (30% compared to 50%) and a more conservative clustering algorithm, we found that the proportion of singleton to nonsingleton genes increased (66% versus 50%), with 77,371,367 singletons and 40,257,814 nonsingletons compared to our previous database (26). This indicates that at lower percent identities, over half of the resulting gene clusters are still singletons, despite a greater number of samples and ecosystem types than in prior analyses.

### Using microbiome genetic fingerprints to estimate the size of, as well as similarities between and within, microbial pangenomes.

Akin to prior efforts working at the taxonomic level, we aimed to compare the similarity of different ecologies based on gene content (i.e., the presence or absence of a gene assembled in a given sample) in an effort to determine, overall, how pangenomically similar different environments are. To control for bias due to sample size and the sequencing depth of individual samples, we iteratively computed the Jaccard distance between each pair of ecologies based on the number of shared genes between four random samples of each ecology (see Materials and Methods). Ecologies segregated, broadly speaking, according to broader environmental context, including gut-associated samples, environmental samples, and nonhuman hosts (Fig. 1B). For example, gut microbiome samples tended to group together (i.e., from chicken, moose, mouse, human, and cow). Analogously, environmentally sampled, non-host-associated microbiomes (e.g., aquatic, aquatic sediment, terrestrial soil) had similar overlapping gene content. Nasal, skin, and vaginal samples also clustered together, while samples from coral reefs and plants shared limited gene content with other ecologies. Plant ecologies, on average, are the least like all other ecologies (Fig. 1B). Samples from human gut, human mouth, and mouse microbiomes were the most similar to other ecologies on average across all sampling iterations.

We found that the number of unique genes in an ecology did not correlate with the sample size for that ecology. Further, to confirm that our results were not an artifact of our method, we performed a permutation test, randomizing sample gene content and ecology, showing that there was limited similarity between the values in the heatmap in Fig. 1B and this randomized data set (Fig. S2).

10.1128/msystems.00118-23.2 FIG S2 Permutation test of data in Fig. 1. Randomizing the gen ecology mapping, we recomputed the subsampling analysis in Fig. 1, comparing the gene content of different ecologies. (A) Hierarchical clustering on the Jaccard distance between ecologies as a function of iterative sampling. Each cell represents the Jaccard distance between two ecologies (in rows and columns) after 50 random samplings. The cell color is in units of Jaccard distance. The color of text corresponds to broader ecology class. (B) Average number of genes unique to (found only in) a given ecology. Download FIG S2, PDF file, 0.4 MB. Copyright © 2023 Zimmerman et al. 2023 Zimmerman et al. https://creativecommons.org/licenses/by/4.0/ This content is distributed under the terms of the Creative Commons Attribution 4.0 International license.

We additionally counted the number of genes that were unique to a given ecology compared to all others (Fig. 1C). A number of environments contained limited numbers of unique genes. These included the human nasal cavity (average unique gene count, 8,670; sample size, 189), human vaginal samples (average unique gene count, 7,906; sample size, 88), human airways (average unique gene count, 15,356; sample size, 137), human skin (average unique gene count, 60,030; sample size, 418), chicken cecum (average unique gene count, 59,655; sample size, 27), and moose gut (average unique gene count, 50,83; sample size, 4). Conversely, the cow gut microbiome had the highest number of genes on average that were unique to any given ecology (average gene count, 470,685; sample size, 24) (Fig. 1C). In other words, the cow microbiome is genetically more “distinct” from all other microbiomes on average, more than the vaginal microbiome, for example.

**FIG 2:**
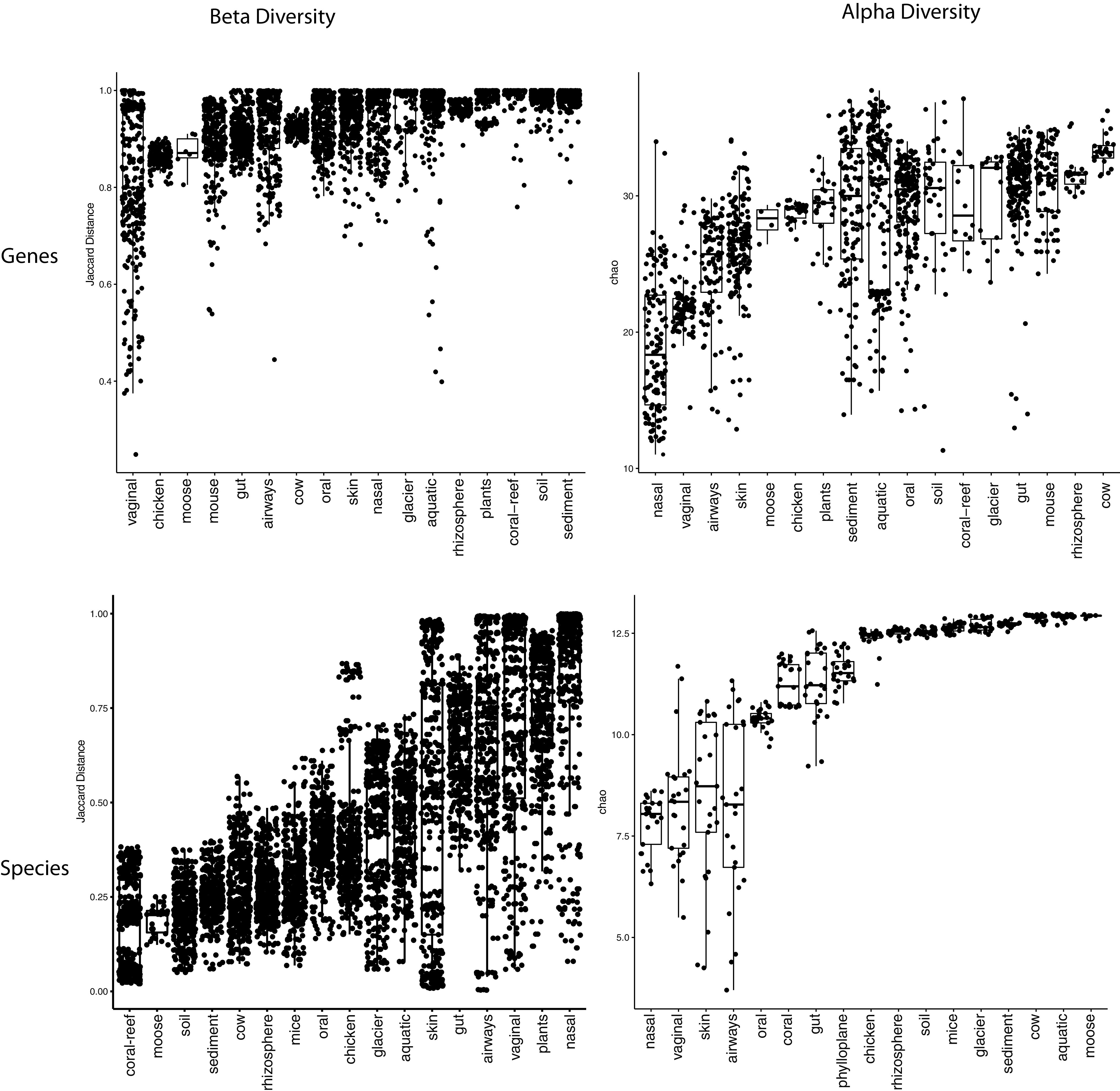
Alpha and within-ecology beta diversity of samples in each ecology. (Top left) Jaccard distance between each pair of samples used in Fig. 1B computed through shared gene content. (Bottom left) Bray-Curtis distance between each pair of samples in each ecology calculated via species abundance from Table S4 in the supplemental material. (Top right) Chao2 richness estimator of each sample used in Fig. 1B. (Bottom right) The Chao1 richness estimator of 4 samples from each ecology from Table S4 calculated via species abundance.

To compare samples within ecologies, we also computed the richness of each sample and difference between samples in the same ecology using both species abundance and gene prevalence (Fig. 2). At the gene level, the nasal microbiome has the highest beta diversity among all human microbiome ecologies but the lowest alpha diversity among all ecologies. In other words, individual nasal microbiomes have a very low number of genes, but pairs of nasal samples are highly genetically dissimilar. This is also reflected at the species level in which the nasal microbiome has the lowest number of species among all microbiomes, but the highest species diversity. The opposite is true for the mouse microbiome, which has high species and gene richness, as well as high numbers of shared genes and species, potentially indicating increased consistency in mouse pangenomes compared to nasal.

10.1128/msystems.00118-23.10 TABLE S4 Pan-ecologically conserved gene metadata. File containing the functional annotations and mapping to GTDB genes for the 1,864 pan-ecologically conserved gene sequences identified in our analysis. Download Table S4, XLSX file, 0.5 MB. Copyright © 2023 Zimmerman et al. 2023 Zimmerman et al. https://creativecommons.org/licenses/by/4.0/ This content is distributed under the terms of the Creative Commons Attribution 4.0 International license.

### Massive-scale dimensionality reduction analysis identifies gene-level ecological similarities.

**FIG 3:**
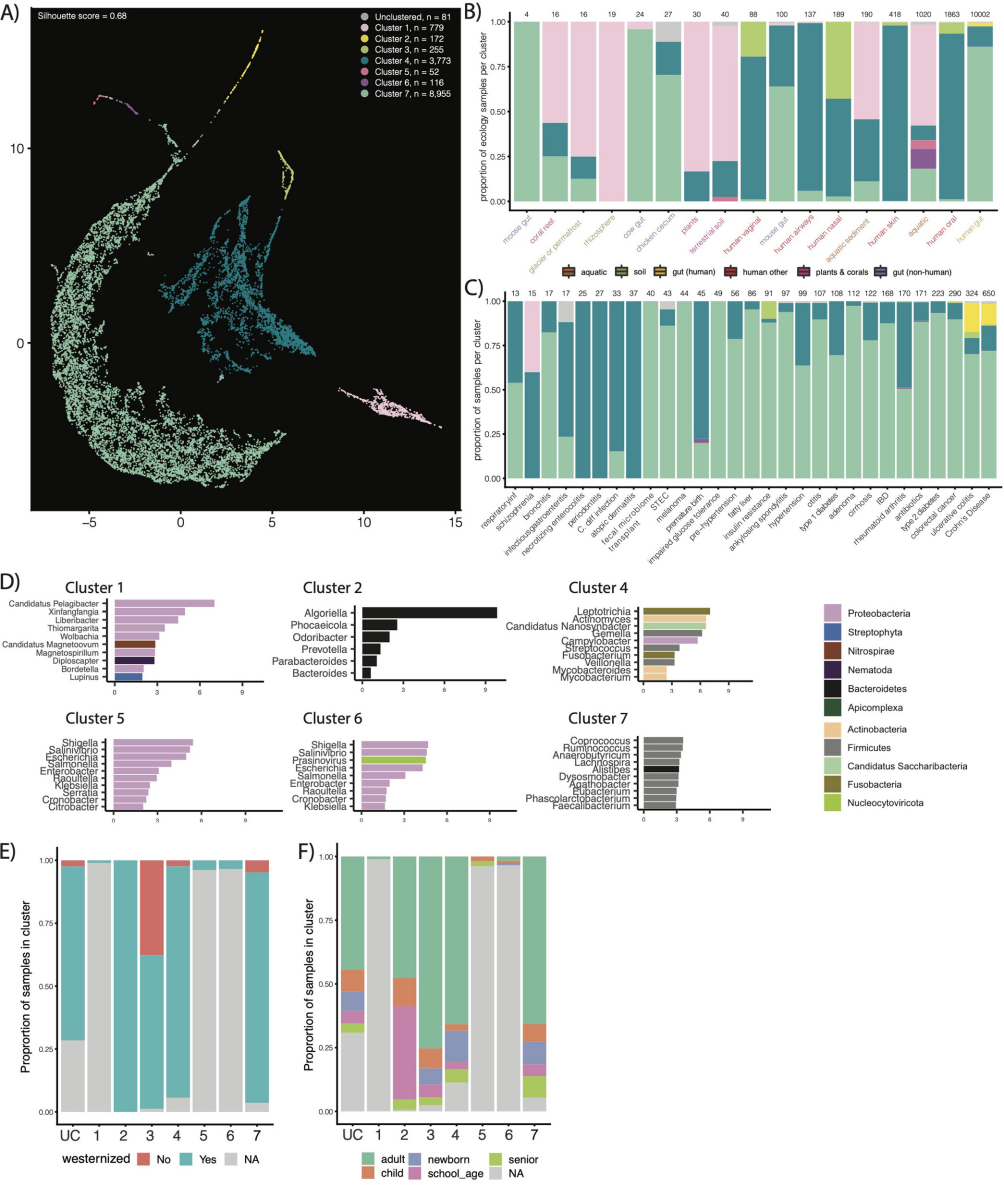
Results of our unsupervised gene-level clustering analysis. (A) Output of our UMAP analysis displaying all seven clusters we identified. Cluster composition corresponds to color. (B and C) Using the same color scheme as in panel A for the bars, the proportion of samples in different ecologies and with different disease states in our identified clusters. (D) Top 10 enriched genera, colored by phyla, in each cluster. (E) Distribution of Westernized versus non-Westernized samples across clusters. (F) Distribution of age categories across clusters.

We next aimed to cluster the 14,183 samples in our database according to the 117 million nonredundant genes captured within them to identify and compare underlying genetic structures across ecologies. We used latent semantic indexing (LSI) to achieve this end (Fig. 3). LSI analysis identified seven “types” of metagenomes, grouped into separate clusters across our data set (silhouette score, 0.68). We found that cluster composition was, on average, robust to parameter choice (Fig. S3).

10.1128/msystems.00118-23.3 FIG S3 Robustness of unsupervised clustering. We show a series of parameters (described in rows and columns) used to generate UMAP output, as well as the number of clusters generated, silhouette score, and number of unclustered samples. The number of neighbors (10, 15, 20, and 30) and the minimum distances (0.1, 0.25, and 0.5) used to construct the UMAPs are provided in the columns and rows. We also varied the minimum size of clusters. UMAPs with minimum cluster size of 50, 75, and 100 can be seen in panels a, b, and c, respectively. The red box illustrates the chosen UMAP seen in Fig. S2. Download FIG S3, PDF file, 2.0 MB. Copyright © 2023 Zimmerman et al. 2023 Zimmerman et al. https://creativecommons.org/licenses/by/4.0/ This content is distributed under the terms of the Creative Commons Attribution 4.0 International license.

Unsurprisingly, we found that ecology dominated cluster composition, and we thereby aimed to compare the ecological makeup of samples within our seven identified clusters (Fig. 3B and C). The majority (99.4%) of the human microbiome samples were contained within four clusters, including clusters 7, 4, 3, and 2. The remaining clusters contained the majority of environmental samples (69.3%), though some environmental samples (mostly aquatic and aquatic sediment) were dispersed among the human ecology-dominated clusters (26.4% and 45.7%, respectively). It is worth noting that smaller clusters tended to arise from samples with lower numbers of genes (e.g., cluster 2 has only 172 samples with about 295 genes per sample) (Fig. S4), indicating that sample size may confound clustering to a certain degree. That said, some of the gene richness from samples may arise from ecology-specific batch effects as well (e.g., nasal microbiomes having fewer genes than human gut samples).

10.1128/msystems.00118-23.4 FIG S4 Potential biases due to depth of sequencing in cluster analysis. Plot depicts the average number of genes across all samples in all seven clusters identified by unsupervised clustering. Download FIG S4, PDF file, 0.07 MB. Copyright © 2023 Zimmerman et al. 2023 Zimmerman et al. https://creativecommons.org/licenses/by/4.0/ This content is distributed under the terms of the Creative Commons Attribution 4.0 International license.

Cluster 2 (172 samples) was entirely (100%) human gut microbiome samples, while cluster 3 contained only nongut (vagina, nasal, and oral) human microbiome samples. Most gut microbiome samples fell in cluster 7 (total, 8,955 samples; human gut, 8,597). The second-largest group of gut microbiome samples lay in cluster 4 (total, 3,773; human gut, 1,137), which also contained the majority of human nongut ecologies (oral, nasal, vaginal, skin, and airways). The gut samples from nonhuman hosts were split among the environmental and human-dominated clusters; however, most of them (73.5%) were also grouped in clusters 7 and 4.

We next compared the phylum- and genus-level taxonomic enrichments within each cluster. We annotated each ORF in every sample according to taxonomy by alignment using the lowest common ancestor (LCA) algorithm. We found all seven clusters have distinct taxonomic and functional signatures (Fig. 3D; Fig. S5). The nongut clusters were dominated by a variety of environmental and human-associated genera. Notably, cluster 3 was enriched in the *Homo* genus and several other genera in the Chordata phylum, indicating contamination (Table S2). The clusters containing samples from the human gut microbiome fell into three categories. Cluster 2 was dominated by *Bacteroidetes* and was notably enriched for *Odoribacter*, *Prevotella*, *Parabacteroides*, and *Bacteroides*. It was additionally notably enriched in transposases. Cluster 4 was not dominated by a single taxonomic clade, containing a variety of oral and gut-associated taxa (e.g., Streptococcus and *Fusobacterium*). Cluster 7 was dominated by *Firmicutes* and *Bacteroidetes*, including a number of well-studied gut genera, like *Faecalibacterium*, *Alistipes*, *Lachnospira*, and *Ruminococcus*.

10.1128/msystems.00118-23.5 FIG S5 Functional enrichment in clusters. The top 25 enriched protein products in each of the seven clusters depicted in Fig. 2. Download FIG S5, PDF file, 0.6 MB. Copyright © 2023 Zimmerman et al. 2023 Zimmerman et al. https://creativecommons.org/licenses/by/4.0/ This content is distributed under the terms of the Creative Commons Attribution 4.0 International license.

10.1128/msystems.00118-23.8 TABLE S2 Genera enriched in cluster 3 of UMAP. This file contains the genera that are enriched in cluster 3 of the UMAP with the corresponding *P* values, *t* values, fold change, and BY-adjusted *P* values of each enriched taxa calculated via a one-sided *t* test. Download Table S2, XLS file, 0.03 MB. Copyright © 2023 Zimmerman et al. 2023 Zimmerman et al. https://creativecommons.org/licenses/by/4.0/ This content is distributed under the terms of the Creative Commons Attribution 4.0 International license.

### Metagenomes segregate genetically according to age and Westernization, but not health status.

Host disease status, overall, did not contribute to cluster membership. No single cluster contained all of the samples with human diseases, nor was one cluster exclusively “health associated.” That being said, a subset of the inflammatory bowel disease (IBD) samples were uniquely relegated to the smallest, *Bacteroidetes*-dominated gut cluster (cluster 2), which was otherwise healthy. Cluster two also only contained (i) Westernized samples, and (ii) nonnewborn samples. This perhaps indicates a link between Westernization status and a subtype of IBD reflected through joint changes to specific microbes. The other human-gut microbiome-dominated clusters, conversely, had a range of samples from all ages and a combination of Westernized and non-Westernized individuals (Fig. 3).

In order to further identify if Westernization and age guided the position of different samples within a cluster, we reran our clustering and dimensionality pipeline on the four gut-dominated clusters (2, 3, 4, and 7) specifically (Fig. S6). We found that samples consistently segregated according to Westernization and ecology; however, the degree to which they did for age was cluster dependent. For example, in clusters 2 and 3, metagenomes did not group according to age. They did, however, in clusters 4 and 7. In cluster 7 specifically, we found newborns to have similar genetic compositions to other, nongut, body sites (e.g., the human oral microbiome).

10.1128/msystems.00118-23.6 FIG S6 Results of subclustering analysis. Rows correspond to the named cluster (with the gray inset showing the original shape of the cluster in Fig. 2). Columns correspond to the color scheme of points by ecology, age, and Westernization. Download FIG S6, PDF file, 3.5 MB. Copyright © 2023 Zimmerman et al. 2023 Zimmerman et al. https://creativecommons.org/licenses/by/4.0/ This content is distributed under the terms of the Creative Commons Attribution 4.0 International license.

### Ecologically contextualizing the gut ecosystem yields a database of discrete and shared gene sets.

**FIG 4:**
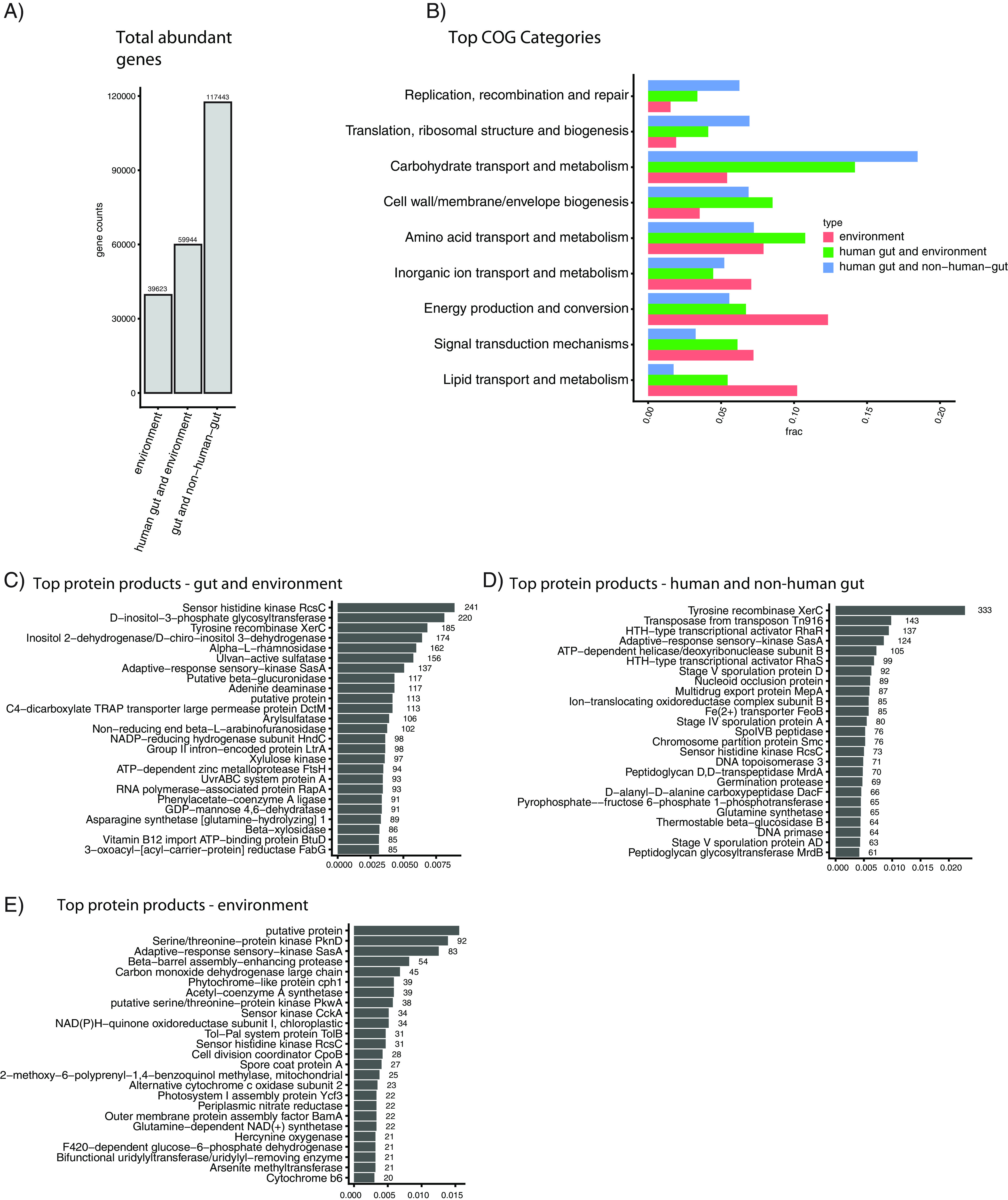
Functional analysis of genes abundant in different ecological contexts. (A) Total number of genes captured in the differential abundance analysis. (B) Fraction of genes in the different high-level COG categories, by intersection. (C to E) The top 25 most common protein products for each ecological comparison considered.

We next aimed to contextualize the gene content of the human microbiome by identifying ecology-specific (e.g., broadly gut-associated) genetic elements of metagenomics. We identified sets of genes that, compared to all other ecologies, were abundant in (i) human gut and environmental samples, (ii) human gut and nonhuman gut samples, and (iii) environmental samples alone. Specifically, we identified genes that were prevalent in these categories in our initial gene catalog and then tested their differential abundances between different environments in an independent set of 422 samples from our ecologies of interest (see Materials and Methods) (Table S3). We identified 59,944 enriched genes in the human gut and environmental samples, 117,443 genes enriched in the human gut and nonhuman gut samples, and 39,623 genes enriched in the environmental samples (Fig. 4A).

10.1128/msystems.00118-23.9 TABLE S3 Metadata for 422 additional samples. The metadata for the samples used in our abundance calculations of genes identified as being prevalent in different ecological groupings. Download Table S3, XLSX file, 0.04 MB. Copyright © 2023 Zimmerman et al. 2023 Zimmerman et al. https://creativecommons.org/licenses/by/4.0/ This content is distributed under the terms of the Creative Commons Attribution 4.0 International license.

**FIG 5:**
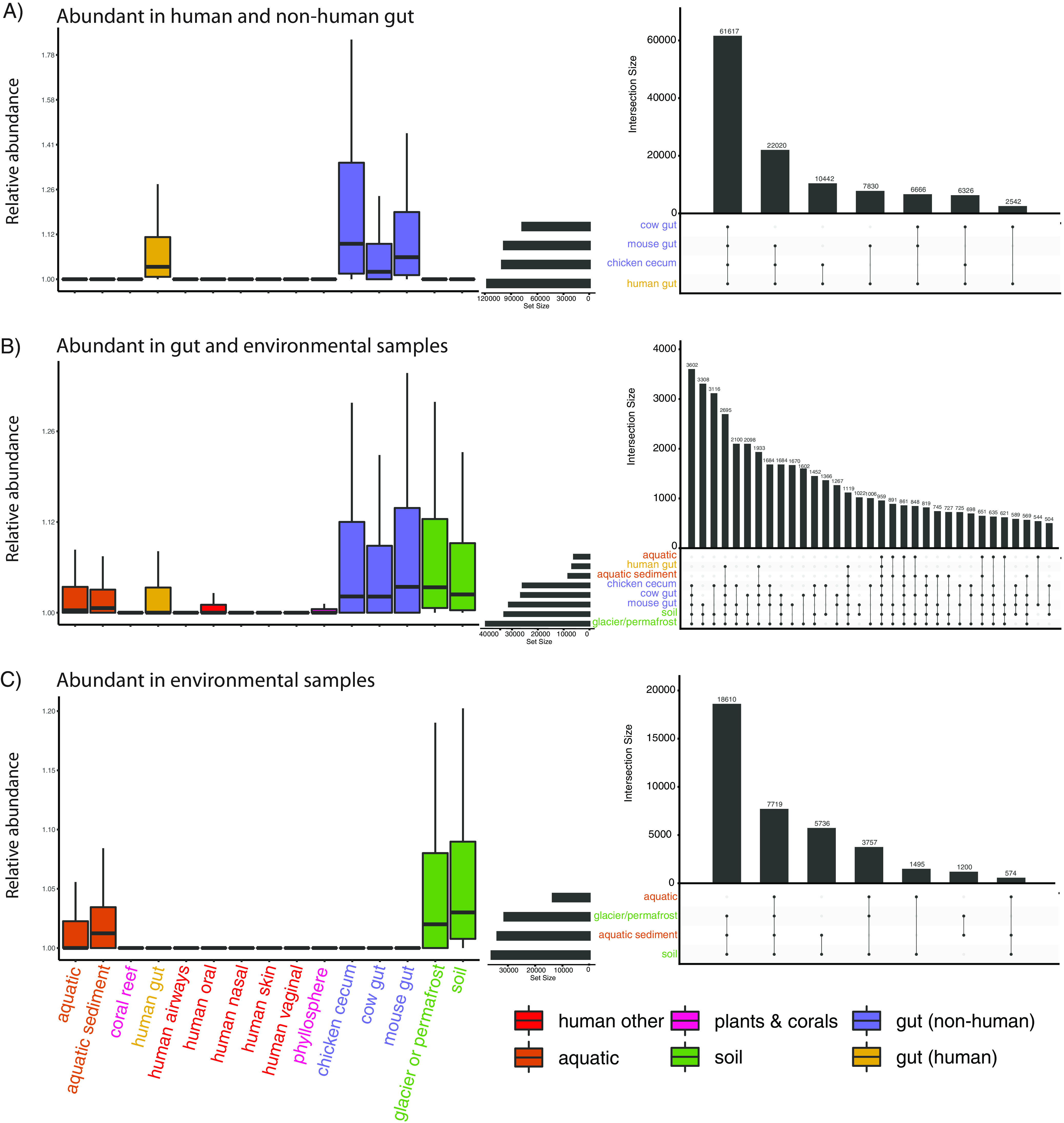
Genes abundant in intersecting ecological groups. (Left columns) Abundance of genes found to be significantly differentially abundant (in a separate cohort) in the described sample types. (Right columns) Number of specific overlaps in abundant genes between specific ecologies.

We found functionally and taxonomically distinct sets of genes in each of the three comparisons we tested (Fig. 5, left column). We additionally explored the specific ecologies in which differential genes overlapped (Fig. 5, right column). For example, the genes overlapping between gut samples and environmental samples were generally found in ecologies containing sediment (e.g., terrestrial soil, aquatic sediment) and nonhuman gut samples. The human gut stood alone (Fig. 5B), generally speaking. Finally, we found that gene abundance in the environmental samples tended to be shared between samples containing sediment, as opposed to purely aquatic samples (Fig. 5C).

We identified the COG categories of each annotated gene, quantifying the proportion of genes in each category (Fig. 4B). Carbohydrate processing was the top category for both human gut and nonhuman gut genes (~20% of all genes with annotations) and human gut and environment genes (~15% of all genes with annotations). Genes abundant in both environmental samples and environmental and gut samples both contained many annotations to the broad categories of energy production and conversion and signal transduction mechanisms.

We additionally analyzed the most common protein annotations in our differentially abundant gene sets, finding that each set contains functionally distinct proteins (Fig. 4C to E). Many of the most common proteins in the environmental category (Fig. 4E) were involved in photosynthesis (e.g., photosystem, cytochrome, and phytochrome proteins) and environmental nutrients (e.g., nitrate reductase, arsenite methyltransferase). Genes abundant in both the gut and environmental ecologies (Fig. 4C) were associated with a variety of nutrient import and export functions (e.g., vitamin B_12_ import ATP-binding protein, BtuD; C4-dicarboxylate TRAP transporter large permease protein, DctM). The top two proteins abundant in human and nonhuman gut ecosystems (Fig. 4D) were both associated with horizontal gene transfer (tyrosine recombinase XerC, transposase from transposon Tn*916*). Other annotations in this category were associated with sporulation (e.g., stage V sporulation protein AD), antibiotic resistance (e.g., multidrug export protein MepA), and cell wall construction (e.g., peptidoglycan glycosyltransferase MrdB, peptidoglycan D,D-transpeptidase MrdA).

### Ecologically contextualized metagenomic architectures identify the genetic features underlying the multiecosystem capability of pathogens.

**FIG 6:**
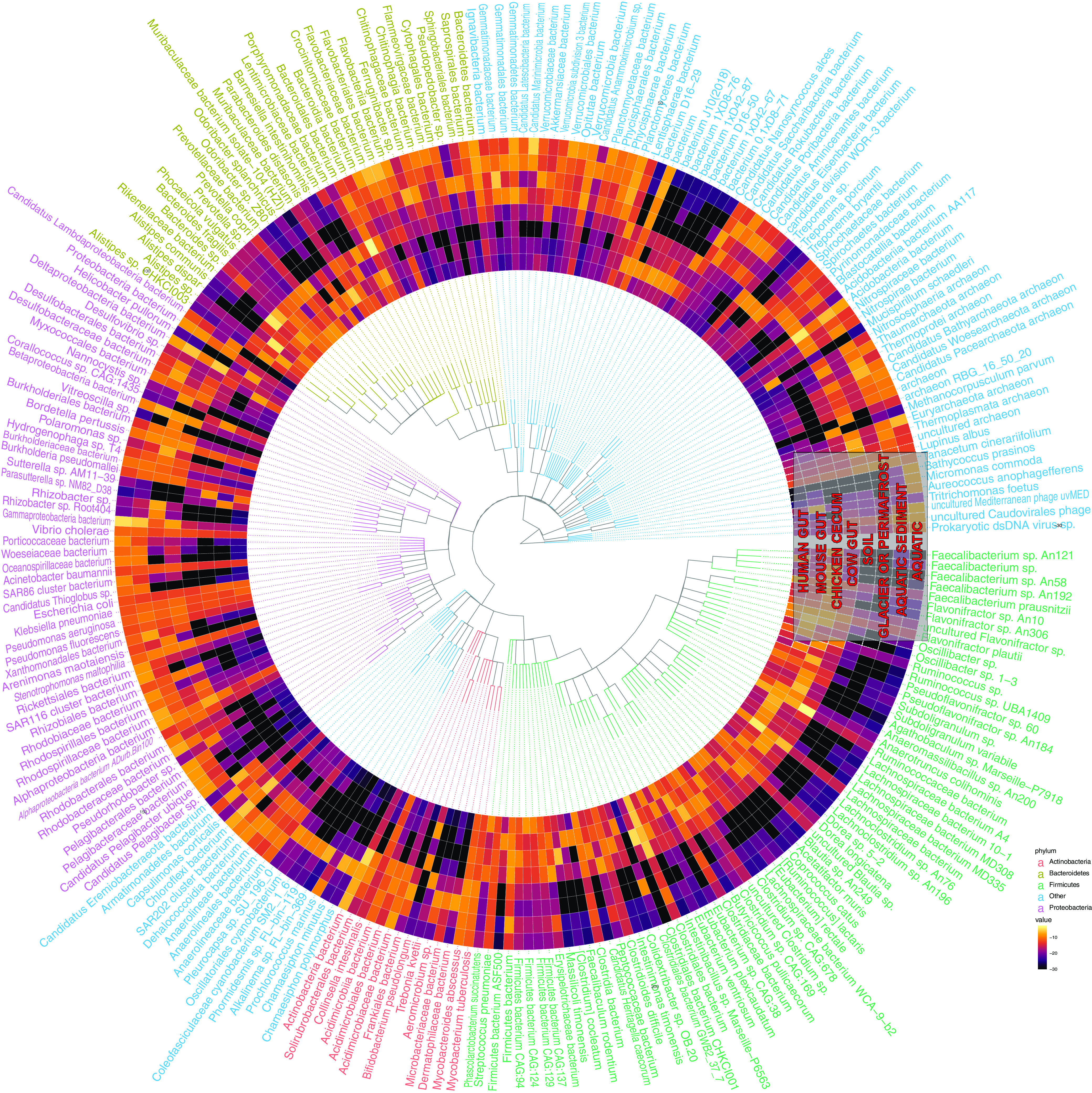
Taxonomically contextualizing the genetic content of the human gut microbiome. Each ring corresponds to a different ecology. Each “row” corresponds to a different taxonomic annotation for an open reading frame that was abundant in any gut or environment microbiome. The colors correspond to the fractions of all genes with a given annotation that were indicated as abundant in a given ecology. Text color corresponds to phyla.

We next sought to identify the taxonomic annotation of genes that spanned environments. We hypothesized that genes found in specific or multiple environments would be those potentially required for an organism to survive in said ecosystems, warranting their further investigation in *in vivo* studies. We compared the most common taxonomic annotations of genes that were abundant in any gut or environmental ecology. First, we taxonomically annotated all open reading frames in all gut and environmental samples using the LCA algorithm in CAT (27). Despite the difficulty of annotating genes to taxonomy (e.g., some annotations, like “*Clostridia* bacterium” being broad and not informative on their own), visualizing the resulting taxonomic bins displayed a clear pattern in phylogeny across human, nonhuman, and environmental ecologies (Fig. 6). Human gut and nonhuman gut samples overlapped predominantly in the *Firmicutes* phylum. Very few *Firmicutes* annotations were highly abundant in environmental samples, relatively speaking. On the other hand, organisms of the phyla *Bacteroides* tended to straddle gut-associated ecosystems and environmental ecosystems. *Proteobacteria* tended to characterize nonhuman gut and environmental samples, with certain organisms, like Vibrio cholerae, having representative genes present across all ecologies. We noted that pathogenic microbes, like V. cholera and Mycobacterium tuberculosis, stood out in the sense that they spanned ecologies.

**FIG 7:**
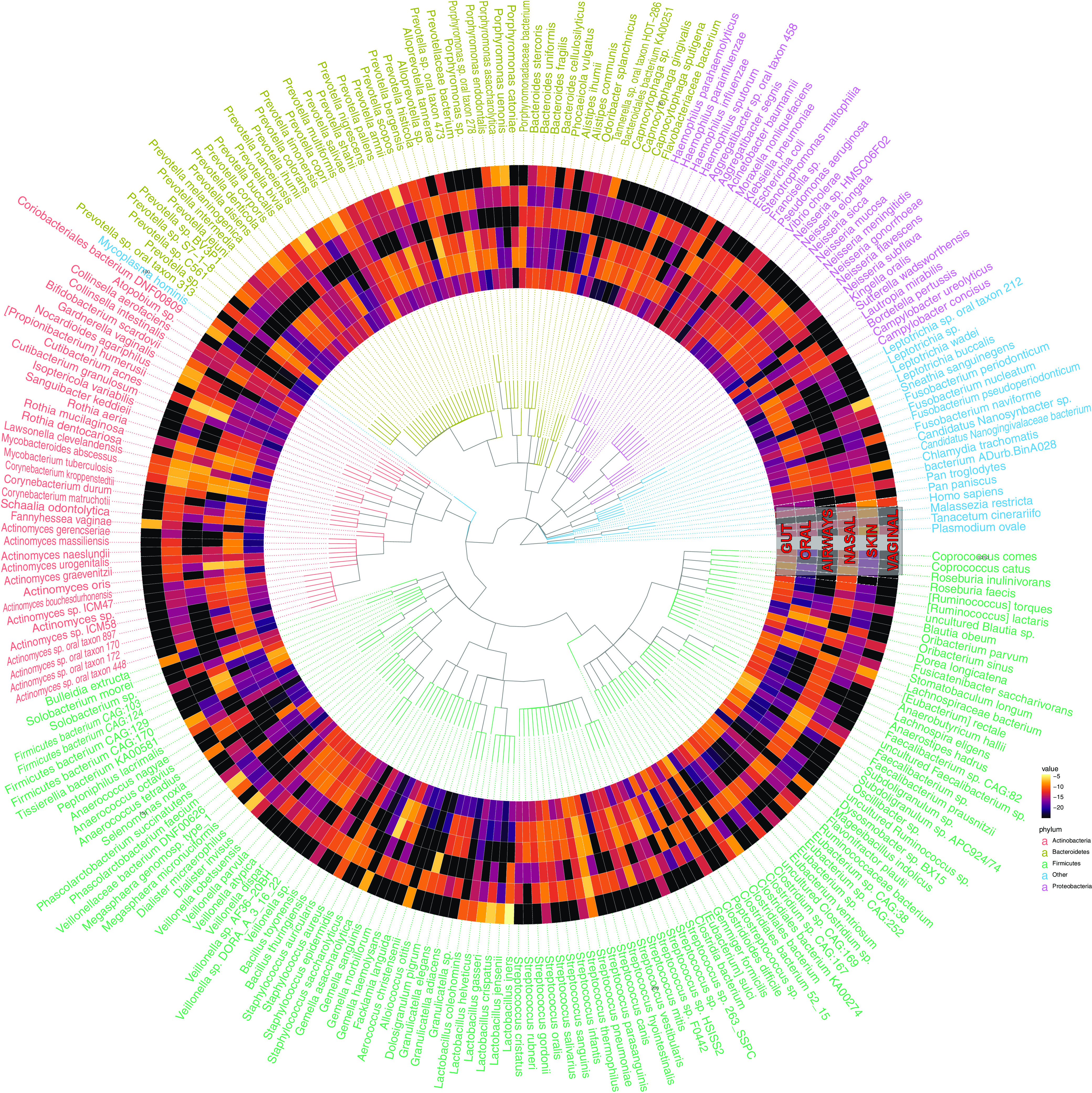
Taxonomically contextualizing the genetic content of the global human microbiome. Each ring corresponds to a different ecology. Each “row” corresponds to a different taxonomic annotation for an ORF in the same cluster as a consensus gene that was abundant in at least two human microbiome body sites. The colors correspond to the fractions of all genes with a given annotation that were indicated as abundant in a given ecology. Text color corresponds to phyla.

Next, we examined the taxonomic distribution of the ORFs in the same clusters as the consensus genes that were differentially abundant in the 6 human-associated ecologies in our data set compared to environmental, plant, and coral ecologies (Fig. 7). The result demonstrated the limited genetic overlap of human microbiome ecologies, notably the lack of shared microbiota with the vaginal and nasal microbiomes. However, we also see that *Clostridia* is shared between the human gut and skin microbiome, while Streptococcus is also shared between the skin, oral, and airway microbiomes, and *Veillonella* is found in the gut, airway, and oral microbiomes. Species that were present in all sites and had many (greater than the mean prevalence of the top 250 species in all ecologies) genes annotated to them, tended, like before, to be potentially pathogenic. These included Staphylococcus aureus, Acinetobacter baumannii, Klebsiella pneumoniae, and Chlamydia trachomatis.

### At least 1,864 genes—not identifiable at high clustering identities—comprise the ecologically conserved elements of metagenomics.

**FIG 8:**
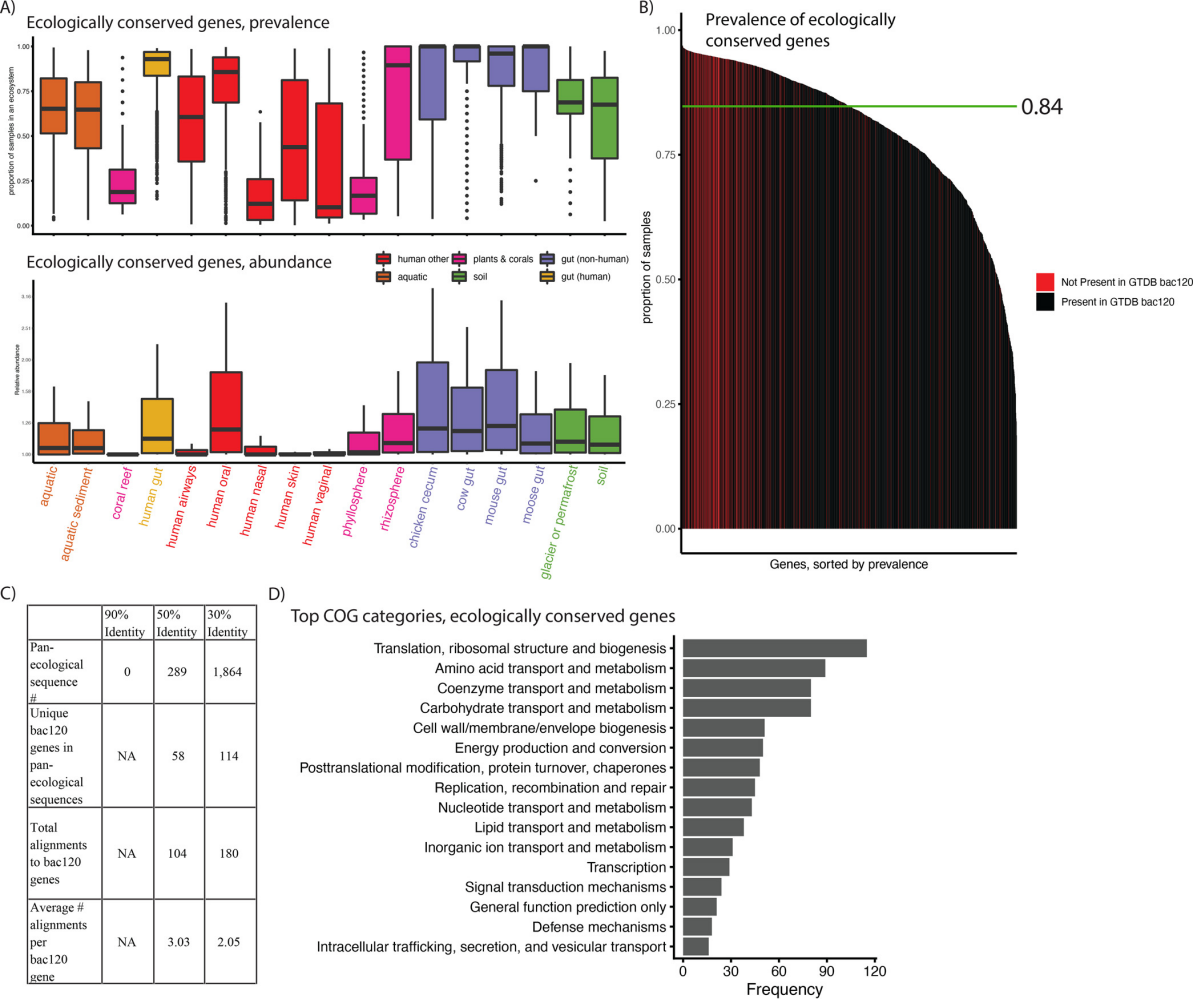
The pan-ecologically conserved genes of metagenomics. (A) The prevalence and abundance (in the 422 external samples used in prior figures), by ecology, of all 1,864 genes found to be assembled at least once in all 17 ecologies. (B) The prevalence of all 1,864 ecologically conserved genes, with colors corresponding to whether or not they aligned to a GTDB bac120 gene. (C) For different percent identities, the number of pan-ecological sequences, the number that align to GTDB bac120 genes, and the average number of bac120 genes aligning to a given identified conserved gene. (D) The most common COG category annotations for the 30% identity ecologically conserved genes.

Finally, we sought to identify sequences that were prevalent and abundant in all 17 ecologies in our database. At 30% clustering identity, we identified 1,864 genes that were found on contigs in at least 1 sample for all ecologies, and we quantified the abundance of the 1,864 genes in the same 422 independent samples from the prior analysis (Fig. 8A, Table S3, and Table S4), finding that both prevalence (median, 0.84) and abundance varied substantially as a function of ecology. We additionally compared their prevalence to that of the bac120 genes used to taxonomically classify bacteria in the Genome Taxonomy Database (GTDB), which are derived from specifically single-copy genes present in at least 95% of a database of microbial genomes (not metagenomes) (28). We hypothesized that searching for globally conserved genes with our approach would be sufficient to recover these genes. We found this to be the case. The bac120 genes had a slightly higher overall prevalence (median, 0.96); however, we were able to identify (via alignment) 114/120 (95%) in our 1,864 genes (Fig. 8B). Notably, the 10 most prevalent genes did not align to the bac120 set (Table S4).

We additionally compared the number of conserved genes found at 30% to those found at the 90% and 50% amino acid clustering identities (Fig. 8C). We found, at 90% identity, that there were no genes found in every ecology, indicating that 90% is too strict a clustering cutoff, as at least the bac120 genes should be identified in the majority of samples. Similarly, we only identified 289 ecologically conserved genes in our 50% gene catalog; however, these aligned uniquely to only 58 of the bac120 genes (48%), and each bac120 gene aligned to an average of 3.05 sequences (compared to 2.03 at 30% identity), indicating that genes clustered at 50% are, on average, “underclustered” for this particular use-case.

We next examined the function of these “ecologically conserved” genes (Table S4). We hypothesized that perhaps they contained functional redundancy and that we were, in fact, referring to genes as “different” when, in fact, they did the same thing. We found this not to be the case. While we did find that the bac120 genes aligned to 180 of out 1,864, indicating mild functional redundancy, we found that, after removing 600 hypothetical proteins, the remaining 1,264 genes were annotated as coding for 1,165 unique proteins.

While, overall, the 1,864 proteins we identified were conserved across microbial life (Fig. 8A), we sought to identify if, specifically, these genes reflected functions not found in the bac120. We found (Fig. 8D) that while the GTDB genes tended to reflect what one would hypothesize to be highly conserved metabolic processes (e.g., ribosome-associated genes), the broader set of ecologically conserved genes did not reveal any consistent set of functions, including a variety of processes from nutrient transport to cell-to-cell signaling. We hypothesize that these may comprise “ecologically core” functions, those not found in every microbial cell but, rather, in all metagenomes.

## DISCUSSION

The genomes of microorganisms that colonize the human ecosystem contain a long evolutionary history spanning many hosts and environments. As a result, to understand the evolutionary and functional dynamics that drive the human microbiome—an ecosystem that modulates so many aspects of human health—we must consider the full ecological spectrum in which microorganisms are found. Our approach here specifically relies on attempting this goal by comparing open reading frame presence or absence in order to genetically contextualize and compare metagenomic life.

We incorporate metagenomes from 17 different ecologies, comparing their genetic content and providing sets of genes that we found to be prevalent and abundant in different ecological contexts, with a specific focus on the gut microbiome. This data set, with its focus on the differential distribution of genes, instead of genomes, complements existing published resources. Further, given that many of its samples are shared between our resource and others, these data sets could feasibly be linked into one metadatabase, where genes can be linked to genomes effectively. For example, our identification of pathogen-associated genes that span environments could be identified in pathogen MAGs and further explored as potential mechanisms of pathogen adaptability to varied environments.

Outside challenges raised with all gene catalog analyses (e.g., suboptimal clustering algorithms) (29), one potential drawback of this analysis is the variation in sample sizes, assembly quality across environments (e.g., corals versus human gut samples), and sequencing depth across environments, which could bias our results in terms of the unsupervised Uniform Manifold Approximation and Projection (UMAP) clustering and the differential abundance analysis. Future work should aim to increase sampling from all metagenomic ecologies in order to fully explore the gene universe of microbiome *writ large*. We additionally note that there is no known “biologically correct” percent identity cutoff for gene catalog analysis, and certainly, some genes likely diverge more than others. Therefore, there is a need for additional algorithmic development that builds functionally motivated gene clusters using different or additional metrics to percent identity alone.

On this note, however, another finding of our report is the utility of clustering at 30% amino acid identity as opposed to the higher levels reported in the literature (30). Clustering based on sequence identity has been contentious in the literature, and clustering at lower percent identities has been computationally infeasible until the development of recent algorithms (29). The inability to identify functionally discrete genes at other clustering identities indicates that prior gene catalog analyses could be improved, or at least better understood, but applying slightly different algorithmic approaches. While we do provide 90% and 50% amino acid identity gene catalogs in our online results, we chose to report the majority of the analysis in this paper using our 30% identity data set. This is because (i) homologous proteins are known to diverge up to 30% identity, and (ii) at least one other database additionally clusters at 30% and reports high-quality clusters with homogeneous functions (11, 31). However, despite the increased stringency of our clustering analysis over past work, we were still able to reproduce a striking prior result, the dominance of singleton genes in metagenomes. When clustering at 30% identity with a more conservative (less greedy) algorithm than before, we still found that singletons, genes assembling in only one sample, comprised >50% of our gene catalog. Given our prior efforts demonstrating that singletons are unlikely to be false-positive genes (26), this implies once more that the gene universe of bacteria *writ large* exceeds what we possibly could have imagined.

Clustering at 30% identity enabled us to identify and characterize 1,864 microbial genes that were ecologically pervasive. We hypothesize, specifically, that these genes represent a class of genetic elements with functions present not in every microbial genome but, rather, in every metagenome, implying perhaps that they carry out functions potentially fundamental to community structure. Further, the fact that these ~1,800 genes did not overlap substantially in function increased our confidence in our choice of a 30% clustering identity. We also showed that these genes would not be detectable using other clustering identities deployed in the literature, 95% nucleotide-level identity (11, 13, 30), compared to a more aggressive threshold, 30% amino acid identity.

Further, the identification of both singletons as well as conserved genes at 30% amino acid clustering identity indicates that prior gene catalog analyses do not even approach capturing the entire genetic diversity of metagenomic systems. We identified 48 million (Fig. 1) genes in the gut microbiome alone, many more than previously observed, and we do expect this number to continue to increase significantly as more humans are sequenced. This calls into question the claims made by prior work that previous sets of microbial gene catalogs of the human gut microbiomes were “close to complete.”(13) Notably, these claims were made using 95% nucleotide identity first with a 3.3-million-gene catalog (30), then again with a 9.9-million-gene catalog (13). It may be that “complete” databases of microbial life, whether they be at the gene or strain level, are infeasible. That said, each of these studies has provided a different window into metagenomic ecosystems, and their expansion, as we attempt to do here, will hopefully continue to do so, whether or not we are able to “completely” sequence the gene content of microbial life.

## MATERIALS AND METHODS

### Overview of approach.

We aimed to compare the gene content of metagenomes across different ecological contexts and provide a database where genes specific to and shared across environments can be queried. We built a nonredundant (nr) gene catalog of 14,183 samples from 462 studies across 17 different ecological contexts. We compared the gene content of each of these ecologies, identifying which are most similar to and different from each other. We then used a dimensionality reduction approach to cluster samples on the genetic level, identifying distinct ecological, taxonomic, functional, and phenotypic features of each cluster. Based on prevalence, we identified the ecologically core elements of metagenomes. We compared these to a database of known conserved genes in bacteria (bac120 from the Genome Taxonomy Database). We additionally used differential abundance analysis on 422 separate samples to identify genes that were abundant in different ecological sets (e.g., in gut-associated biomes). We provide a database of our results at http://www.microbial-genes.bio.

### Data aggregation.

We aggregated publicly available data from a variety of sources, including already published, assembled metagenomes as well as raw shotgun sequencing read data. A full list of data used and summary statistics (e.g., number of genes per sample) are presented in Table S1 in the supplemental material In total, after filtering for samples with low numbers of genes (see next section), we aggregated 14,183 samples from 462 different data sets/studies.

For the human-associated samples, we aggregated assembled samples from our prior work (26) and also one published database that contained assembled contigs (but not predicted genes) (15). This ended up yielding 12,697 samples from 6 different human body sites and 65 studies.

For the environmental and nonhuman host samples, we aggregated data from the MGnify database (https://www.ebi.ac.uk/metagenomics/). We selected all nonhuman host-associated and environmental-assembled metagenomes and accessed their download links. We then manually curated the ~2,200 samples (accessed in winter 2020), removing (i) duplicated assemblies, and (ii) samples where we could not clearly confirm the source of the metagenome by identifying the publication or raw upload data. We found a substantial number of duplicated and/or misannotated samples, so our final sample tally came to 1,486 samples from 397 data sets from 11 ecologies.

### Gene catalog construction.

For the 1,694 samples that we assembled ourselves (i.e., were not already assembled by others), we removed adapter contamination using bbduk, from bbtools v37.62, removed contamination from the human genome with bmtagger v3.101, and carried out *de novo* assembly with MetaSPAdes (32) v3.13.1 using the default parameters. When MetaSPAdes failed due to a currently unresolved bug, we used MEGAHIT v1.2.9 (parameters, –mem-flag 2) (33). We measured *N*_50_s with assemblies using MetaQUAST v5.0.2 (parameters, –max-ref-number 0 -t 15 –fast -m 0). Note that there were 6 samples that, after we downloaded them and included them in our database, became inaccessible (e.g., https://www.ebi.ac.uk/ena/browser/view/ERZ781377), and as a result, we were unable to compute *N*_50_s for them. However, all of these samples had high gene counts (>1,000) and originated from ecologies for which we had many samples (aquatic, cow, mouse), and as a result, we are not concerned about assembly quality or their effect on our results.

To predict open reading frames (ORFs) from assembled contigs, we used Prokka (34) v1.14.6 (parameters, –force, –addgenes, –metagenome, –mincontiglen 1). The functional annotations (e.g., protein names, COGs) in the manuscript are those automatically generated by Prokka. We removed samples that contained fewer than 100 genes. In our prior database, we removed genes <100 bp long; however, given recent work by other teams indicating the physiological import of such short sequences, we chose to leave them in our study (35).

We then clustered all open reading frames predicted by Prokka using the linclust (36) and cluster functions from the MMseqs2 (37) package. As in previous work (26), we initially iteratively clustered (using linclust) our open reading frames, starting at 100% amino acid identity and decreasing, in increments of 5%, down to 30% identity (other parameters, -c [alignment coverage] 0.9). In order to further increase the clustering of the gene catalog we used for the majority of analysis in this paper, we used the more conservative cluster function (same parameters as before at 30% identity) in order to reduce the total number of genes in our 30%, 50%, and 90% catalogs. We chose to use the 30% gene catalog for our analysis due to (i) the use of a 30% cutoff in the literature, and (ii) the fact that many genes, even those involved in core processes, contain homology at near the 30% level (11, 31). We provide these 50%, 90%, and 30% gene catalogs in our resource.

### Gene-level taxonomic annotation.

We assigned best-possible taxonomic annotations to each gene of interest using the built-in least common ancestor computation tool of the Diamond aligner accessed through the CAT software (27, 38). In doing so, we aligned our 30% gene catalog back to the nr reference database and NCBI taxonomy data, annotating, where possible, the NCBI taxonomy of each gene.

### Comparative subsampling of metagenomic ecologies.

In Fig. 1B and C, we used a subsampling strategy to estimate the overall genetic similarity between different ecologies. This was to control for variation in sample size and sequencing depth, as the ecologies we sampled had radically different numbers of observations (e.g., a minimum of 4 moose samples to a maximum of 10,002 human gut microbiome samples). Over 50 iterations, we took 4 samples from each ecology. We made a presence-absence matrix of each gene in each ecology and calculated the Jaccard distance between each ecology. We repeated this process 50 times and averaged the Jaccard distance between each pairwise set of ecologies, as well as the average number of genes that were unique to each ecology. We additionally took this approach for our permutation test (Fig. S2), which we did as a point of comparison, randomizing the gene sample ecology mapping.

In Fig. 2 to calculate the gene-level Jaccard distance within ecologies, we took the same 4 samples from each ecology as described above. We created a presence-absence matrix of each gene in each sample and calculated the Jaccard distance between each sample. We repeated this process 50 times and created a boxplot of the Jaccard distances between each pair of samples in the same ecology. To calculate alpha diversity, we took the same presence-absence matrices and calculated the richness of each sample using the Chao2 estimator.

To calculate species-level beta diversity, we used our previous set of 422 samples to estimate gene abundance (Table S3). Over 50 iterations, we took 4 samples from each ecology and calculated the abundance of each species in each sample using Kracken2 and Bracken (39, 40). For each abundance matrix, we then calculated the Bray-Curtis distance between each pair of samples from the same ecology as well as the richness of each sample using the Chao1 estimator. All alpha and beta diversity measurements were made using the fossil package in R v0.4.0.

### Gene-level dimensionality reduction and clustering of samples.

We aimed to cluster samples based on gene presence-absence using a series of dimensionality reduction approaches. This is a computationally challenging problem due to the massive dimensionality, as it is a sparse matrix with 15 million genes and 14,183 samples. To address this, we performed latent semantic indexing (LSI), a dimensionality reduction method used in other fields like single-cell transcriptome sequencing (RNA-Seq) (41). In this approach, we first performed term frequency-inverse document frequency (TF-IDF) normalization on a binary presence-absence matrix. This is a two-step process where we first divide the presence or absence of a gene (1 or 0) by the total number of genes in each sample. This is called the term frequency and normalizes by the number of genes per sample to make each sample comparable with each other. Next, we calculate the inverse document frequency, which is the log of the number of samples (plus 1) divided by the total occurrences of each gene in all samples. This effectively increases the weight of rarer genes that may distinguish a sample from others. Last, we use matrix multiplication to multiply TF by IDF. This procedure normalizes across samples to correct for differences in gene number and across genes to give higher values to more rare genes.

In the second step of LSI, we input this matrix into a singular value decomposition (SVD) to reduce the dimensionality of our data set into 10, 50, or 100 principal components. We then used UMAP (42) to further reduce our output to two plottable vectors. UMAP was run on each data set 12 times with a combination of different parameters specified below. Reducing the output to two vectors with UMAP facilitates clustering by making the density of samples in a data set more evident. This allows density-based clustering algorithms such as DBSCAN to more easily cluster data. Next, we ran DBSCAN on each UMAP output four times, varying the parameter that specifies the minimum number of samples in a cluster. The clustering results that maximized silhouette score while minimizing the number of unclustered samples were used for downstream analysis and interpretation (Fig. S3).

As described above, we tried a number of different parameters for the SVD, UMAP, and DBSCAN components of our clustering pipeline in order to test the robustness of our clusters as well as select the “best” parameters (SVD, 10, 50, and 100 single values [SV]; UMAP, n_neighbors = 10, 15, 20, and 30, and min_dist = 0.1, 0.25, and 0.5; and HDBSCAN, minPts = 25, 50, 75, and 100). We used the silhouette score and number of unclustered samples to optimize the parameters. We found that the following parameters optimized silhouette score and number of clustered samples (SVD, singular values = 50, UMAP, n_neighbors = 10 and min_dist = 0.1; HDBSCAN, minPts = 50 and n_components = 2) and that the clusters were robust to variation in parameters (Fig. S3). R v4.0.3, package RSpectra v0.16, was used to perform SVD. R version 4.0.2 was used to do the UMAP and clustering. R packages umap v0.2.7.0 and dbscan v1.1-6 were used to do the clustering.

### Dimensional reduction of UMAP gut-associated clusters.

After clustering all samples, we performed additional latent semantic indexing solely on the samples in the clusters containing human gut microbiome metagenomes (clusters 2, 3, 4, and 7). As described above, we first used TF-IDF followed by SVD on the gene presence-absence matrices. Then, we used UMAP to reduce our output into a two-dimensional plane. The parameters used for reclustering all subclusters were SVD, SV = 50; UMAP, n_neighbors = 10 and min_dist = 0.1; and HDBSCAN, minPts = 50 and n_components = 2.

### Cluster-based functional enrichment analysis.

We used the functional annotations of the consensus genes in our 30% amino acid identity catalog to identify those that were enriched in samples present in each of the 7 clusters identified by our prior clustering analysis. To achieve this end, we used the chi-square test, populating a contingency table with the number of genes that have the protein name of interest within a cluster and outside a cluster as well as the overall number of taxonomic and functional annotations within and outside a given cluster. Enriched samples were those with a BY-adjusted *P* value of <0.05 (43). Fisher’s exact tests were used when an element of the contingency table had less than 6 observations.

### Cluster-based taxonomical enrichment analysis.

We used the taxonomical annotations of the open reading frames in our 30% amino acid identity catalog to identify taxa that were enriched in samples present in each of the 7 clusters identified by our prior clustering analysis. To achieve this, we computed the number of genes with a specific taxonomic annotation in each sample. Then, we used a one-sided *t* test to compare the average number of genes annotated to a taxon in one cluster to the average number of genes annotated to a taxon in all other clusters.

### Identification of ecologically conserved genes and differentially abundant genes.

We identified ecologically core genes by finding those that were present at least once in each of our 17 ecologies.

We used both prevalence and abundance to also find genes that were (i) shared between the gut microbiome and other nonhuman gut microbiomes, (ii) shared between the gut microbiome and terrestrial/aquatic microbiomes, and (iii) shared between terrestrial and aquatic microbiomes only. We took (i) genes prevalent in at least one sample of the ecologies of interest but (ii) not present in any other samples, and (iii) those that had statistically significantly higher abundance than all other samples (BY-adjusted *P* < 0.05).

### Comparison of ecologically conserved genes to the GTDB bac120.

We gathered the human Markov models (HMMs) corresponding to the 120 GTDB bac120 genes in Parks et al. (14) from Pfam (v33.1) and TIGRFAM (v15.0). We used hmmsearch (HMMER v3.3.1) to query the 120 HMMs against genes present in all ecologies (1,864 and 289 genes from the 30% and 50% gene catalogs, respectively). Then, we counted the number of HMMs that significantly aligned to the consensus genes (E value < 0.001 and domain E value < 0.001). Hmmsearch parameters are –noali, –tblout <out_file>, –domtblout <out_file>, -E 0.001, –domE 0.001.

### Identification of the taxonomic annotation of genes that span environments.

To identify the taxonomic annotation of genes that spanned the environment, we annotated every raw open reading frame using the LCA algorithm built into CAT v5.2.3 (27). Next, we calculated the frequency of each species-level annotation in each sample.

For species in gut and environmental samples (Fig. 6), we only analyzed species present in at least one gut or one environmental sample and calculated the total number of genes annotated to each species in each environment. To ensure a species was not overrepresented due to increased sample size or sequencing depth of a specific environment, we divided the frequency of each taxon in each ecology by the total number of genes found in the ecology.

For species with greater abundance in the human microbiomes (Fig. 7), we retrieved the open reading frames within the clusters of genes that were enriched in the human microbiome ecologies. We annotated each open reading frame using the LCA algorithm as done above and calculated the number of genes annotated to every species. As above, we then normalized the frequency of species by the total number of open reading frames in each ecology.

To create the actual heatmaps in Fig. 6 and 7, we used ETE3 to create the phylogenetic trees, as well as ggtree and gheatmap functions from the ggtree package v3.2.0 (44).

### Software availability.

The figure generation code is located in our GitHub repository, located at https://github.com/kosticlab/pan_ecological_gene_universe.

### Data availability.

Data generated in this study is hosted by figshare and accessible at http://www.microbial-genes.bio. This resource additionally includes files to ensure the reproducibility of our results, specifically the data needed to run the figure generation code in our GitHub repository (see “Software availability”). A Strengthening The Organizing and Reporting of Microbiome Studies (STORMS) checklist (45) is available at https://figshare.com/articles/dataset/STORM_checklist/20293746.

## Supplementary Material

Reviewer comments
