# Supplementary figures and images for: Quantifying Shared and Unique Gene Content across 17 Microbial Ecosystems
Source: mSystems. 2023 Apr 6;8(2):e00118-23. doi: 10.1128/msystems.00118-23 (PMC10134805; doi:10.1128/msystems.00118-23)

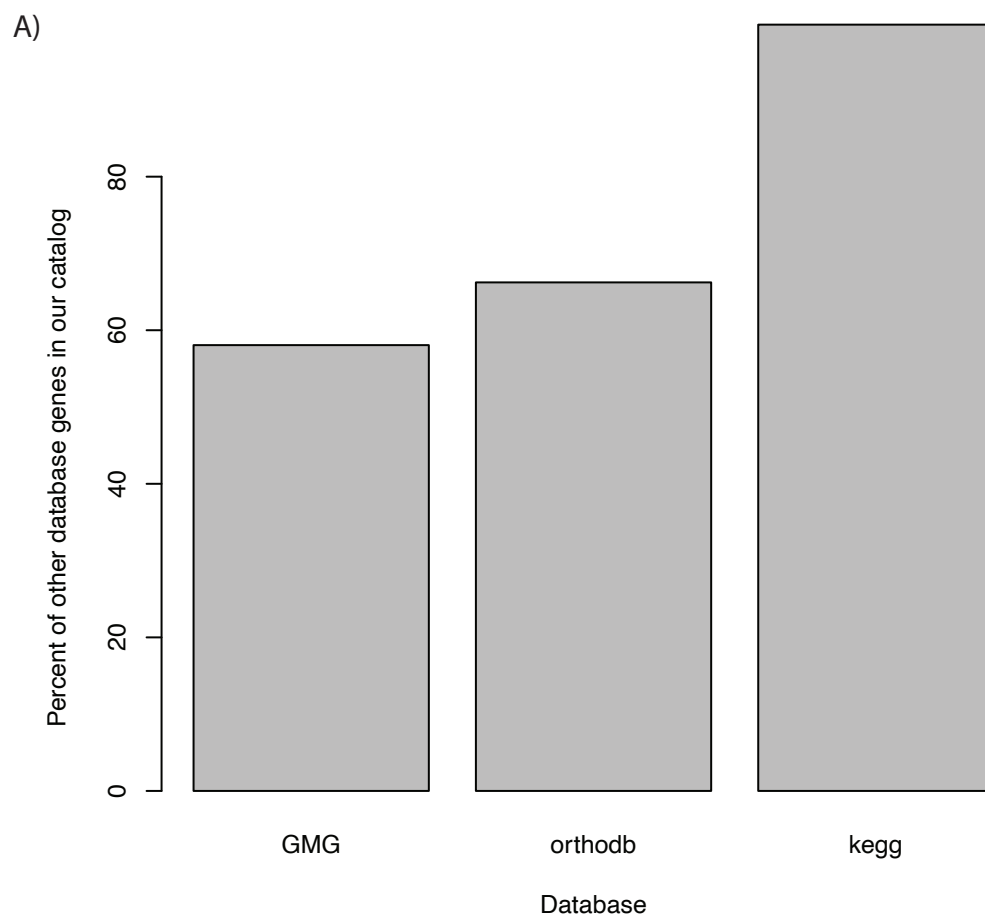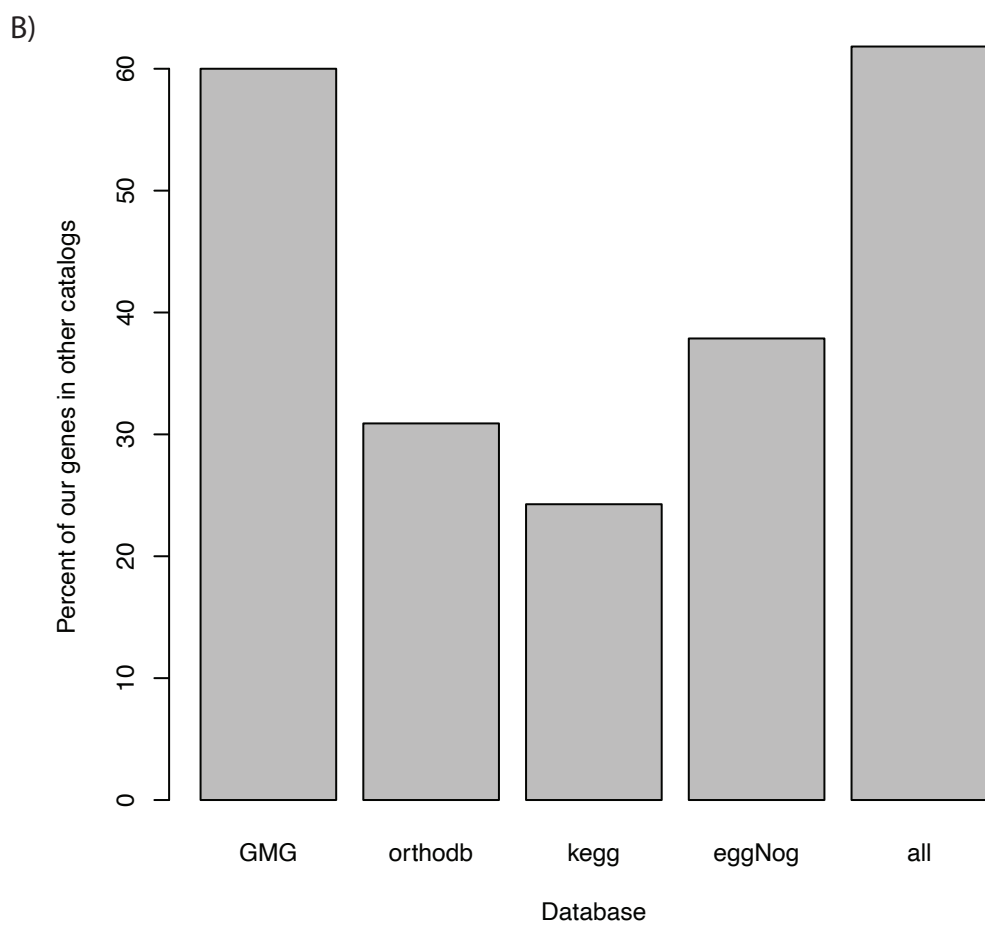

Supplement: FIG S1 [file msystems.00118-23-s0001.pdf]

A)

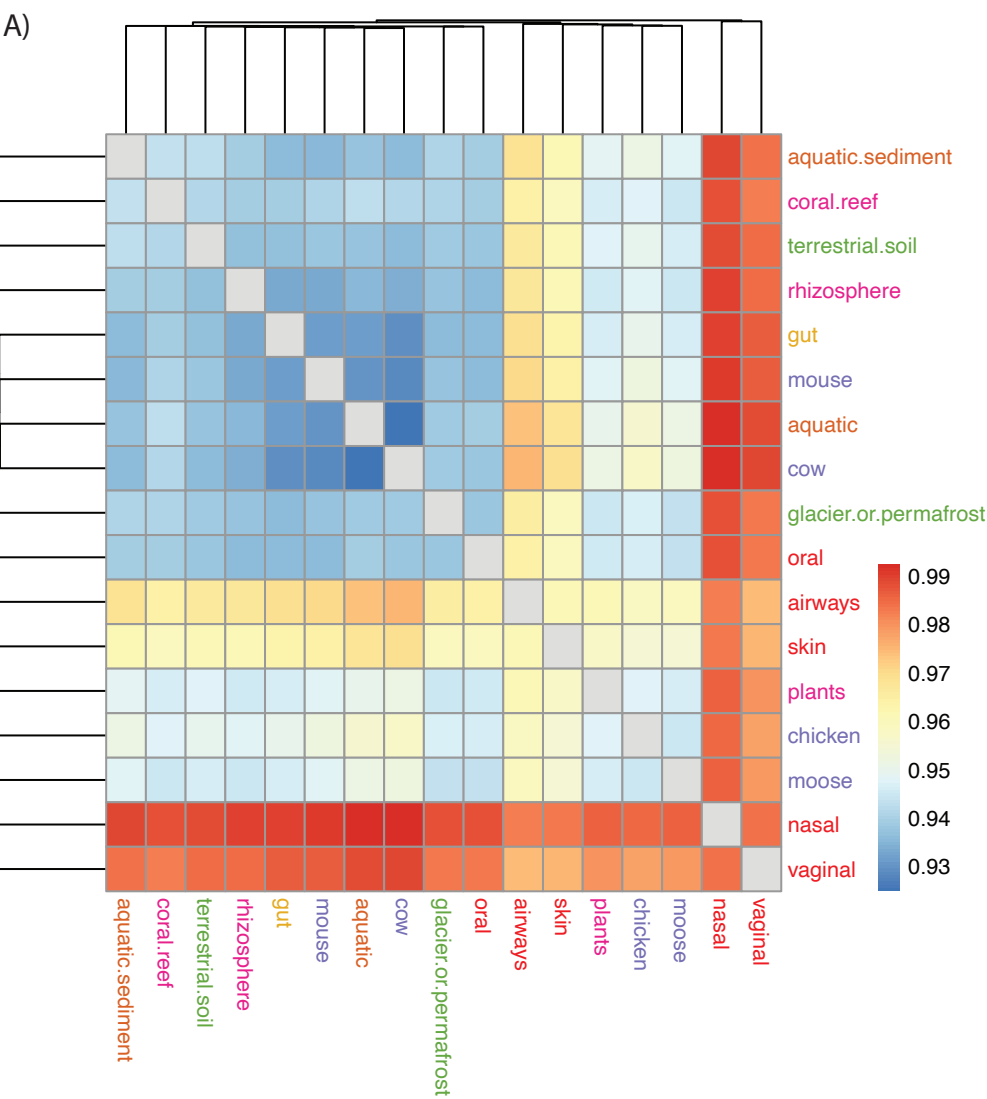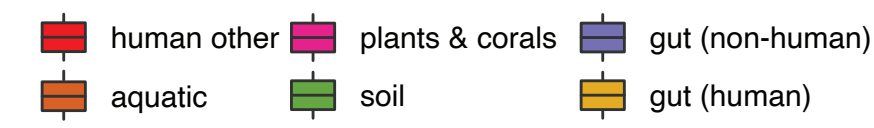

B)

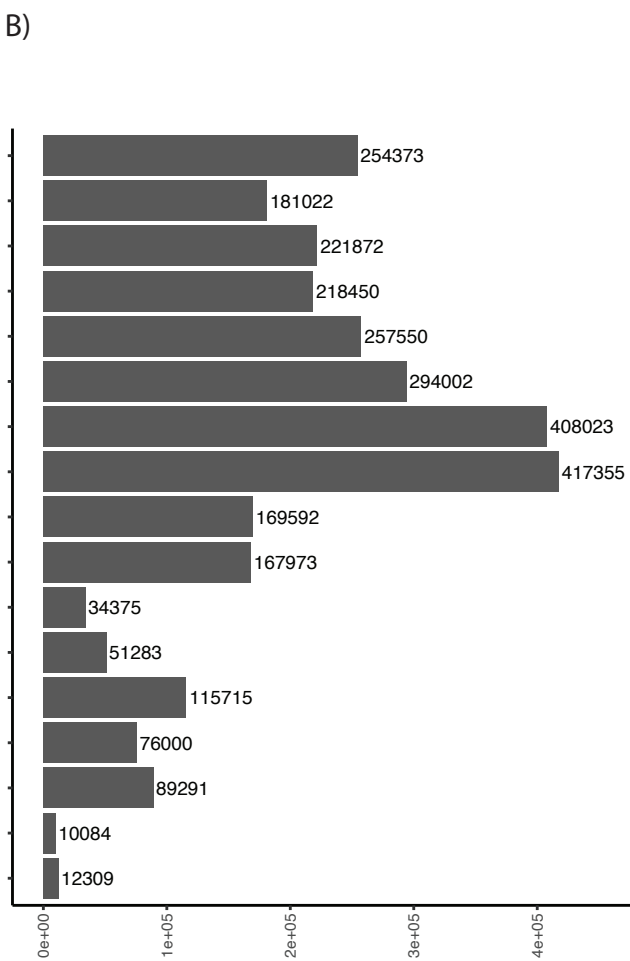

Supplement: FIG S2 [file msystems.00118-23-s0002.pdf]

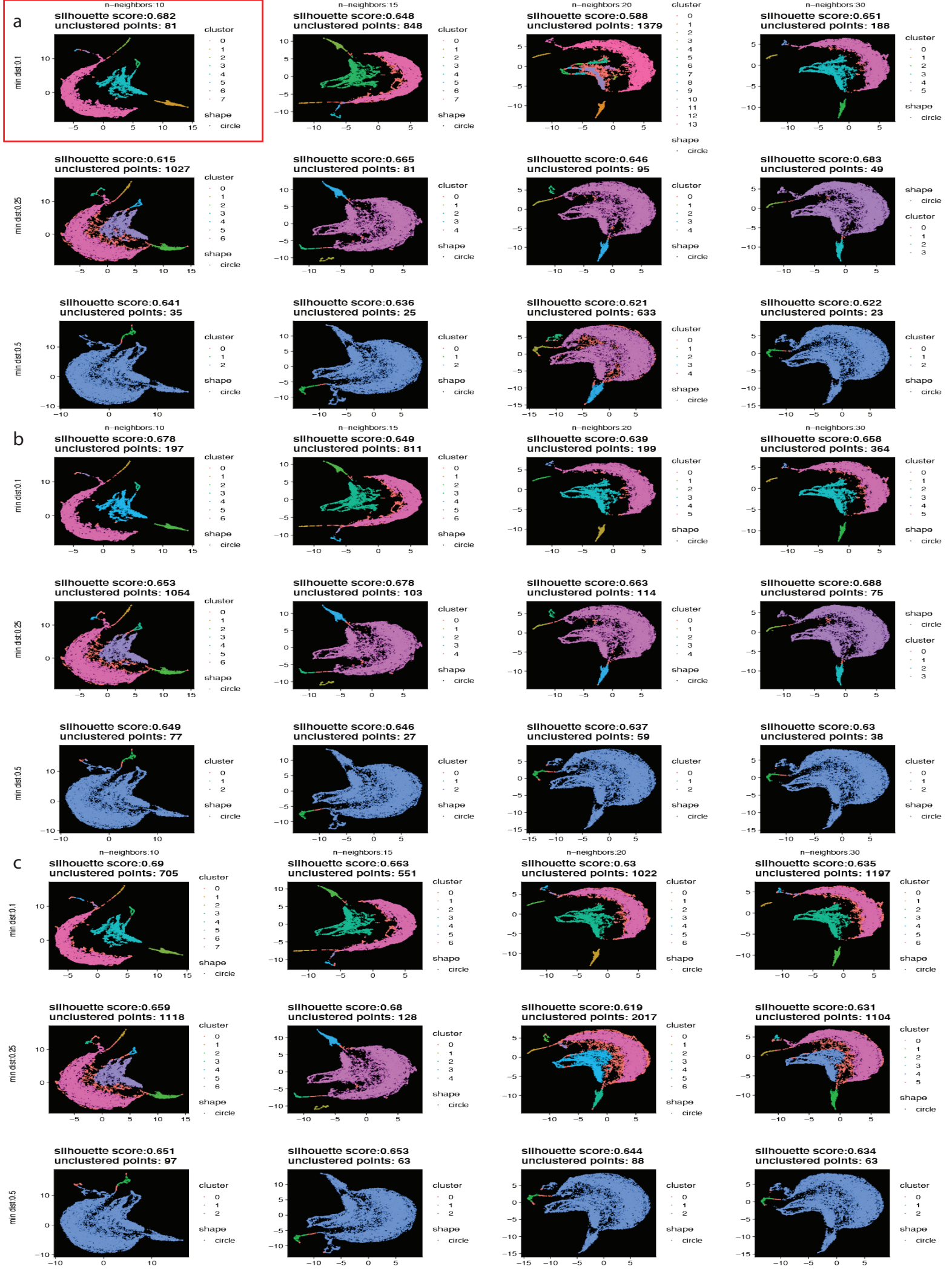

Supplement: FIG S3 [file msystems.00118-23-s0003.pdf]

Average number of ORFs per sample in each cluster

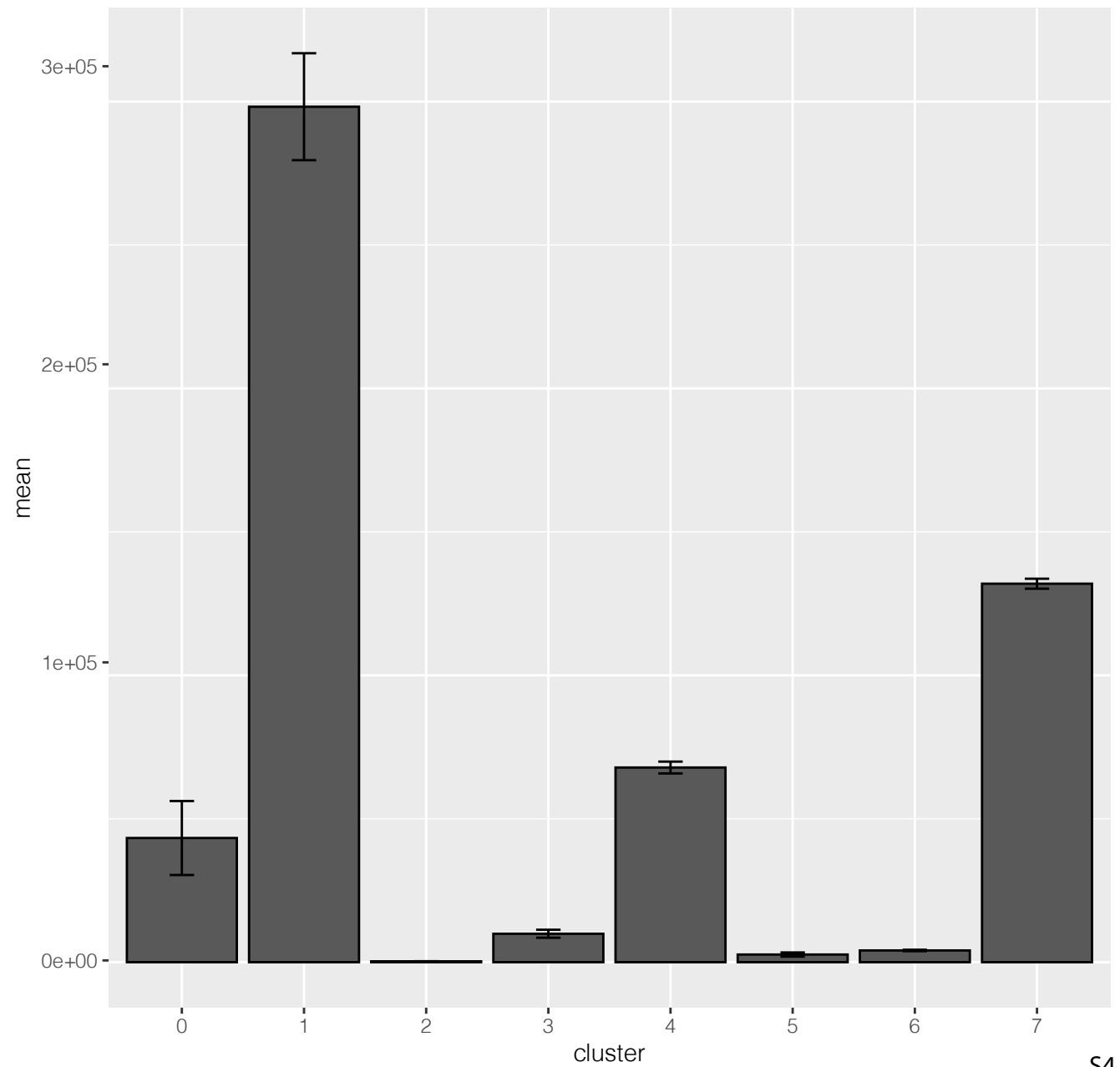

Supplement: FIG S4 [file msystems.00118-23-s0004.pdf]

## Cluster 1

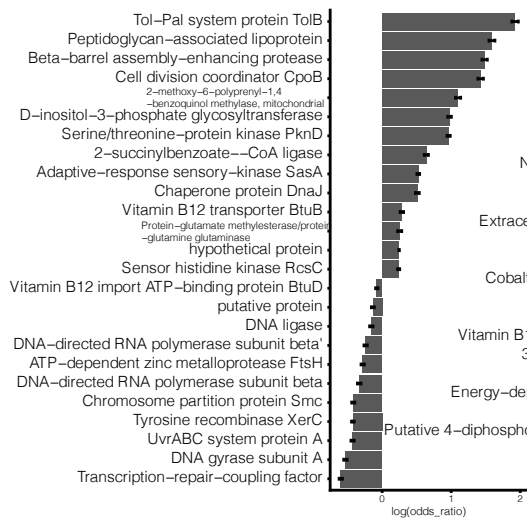

## Cluster 2

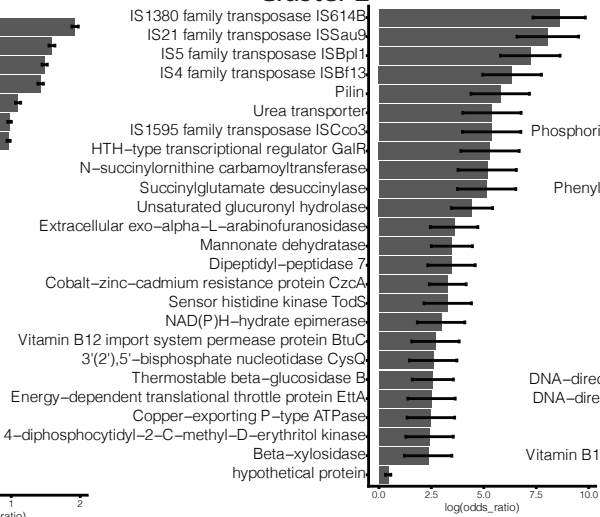

## Cluster 3

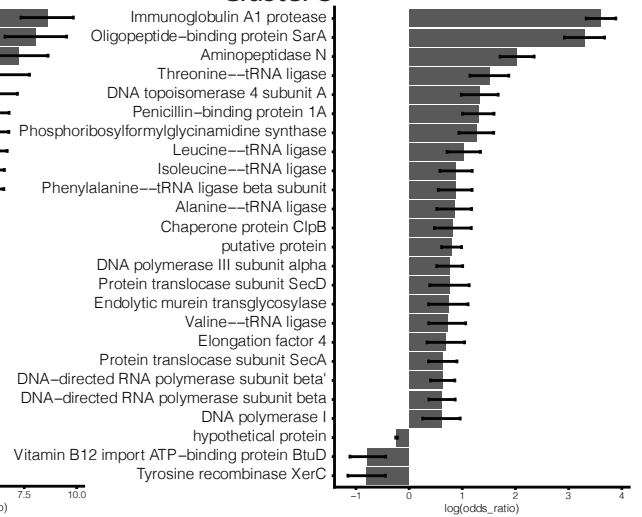

## Cluster 4

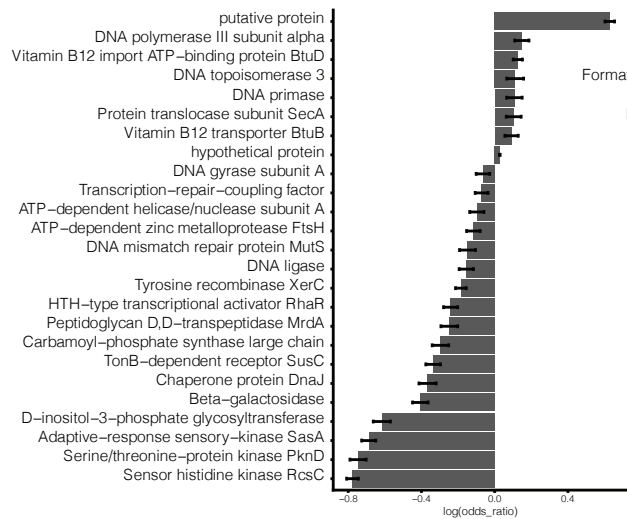

## Cluster 5

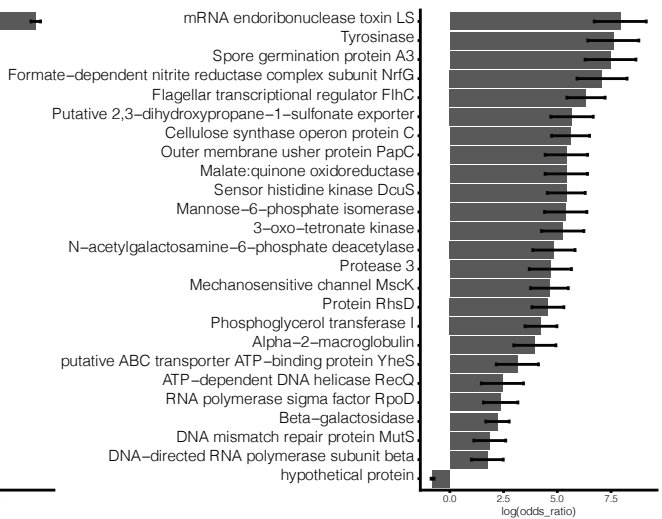

## Cluster 6

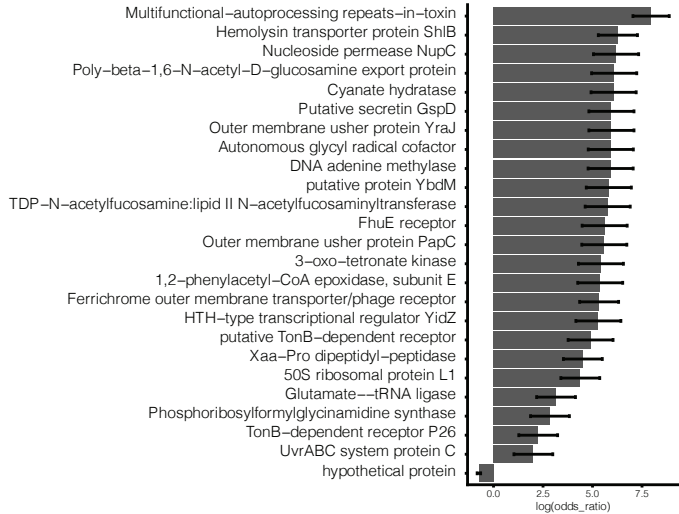

## Cluster 7

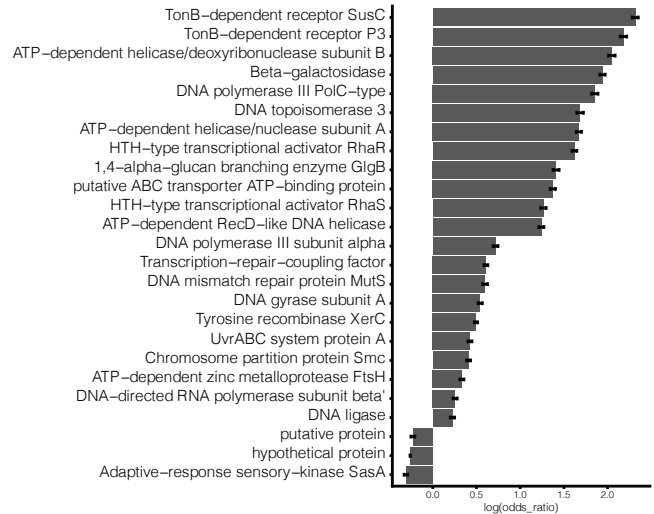

Supplement: FIG S5 [file msystems.00118-23-s0005.pdf]

Ecology

Age

Westernization

Cluster 2

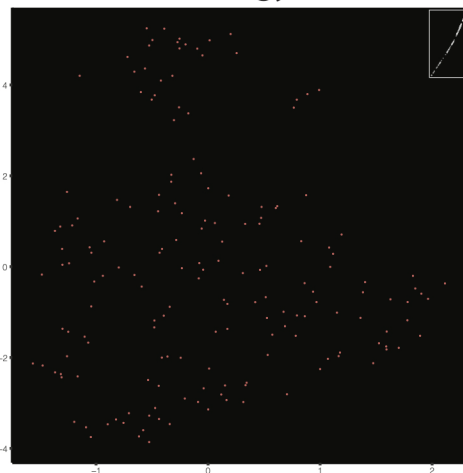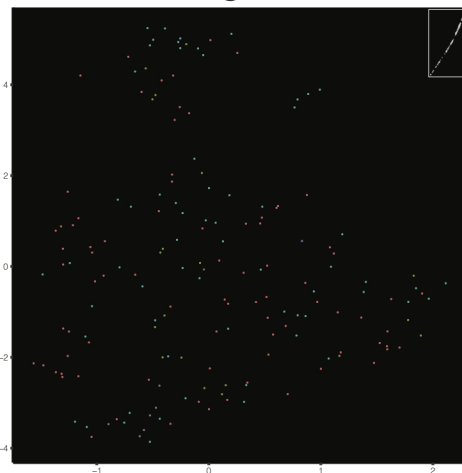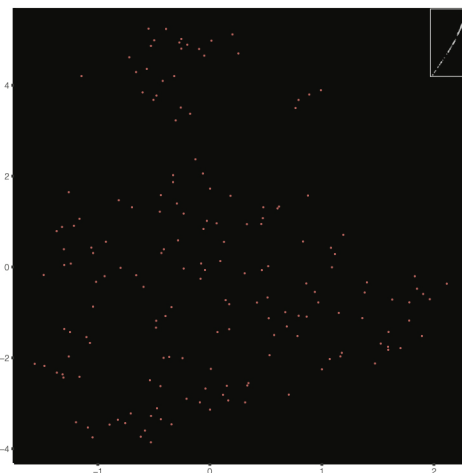

Cluster 3

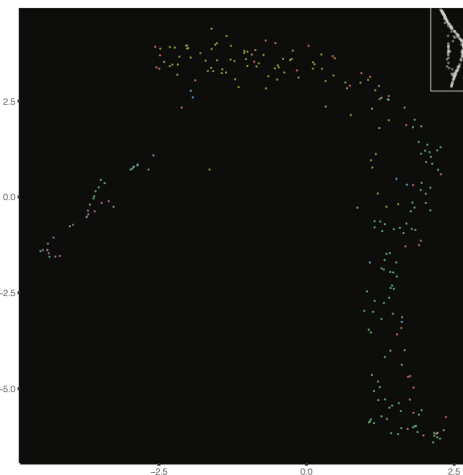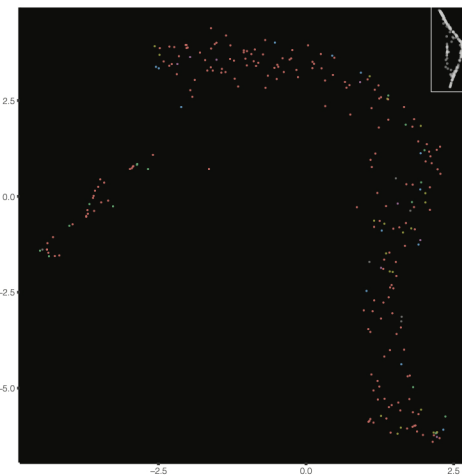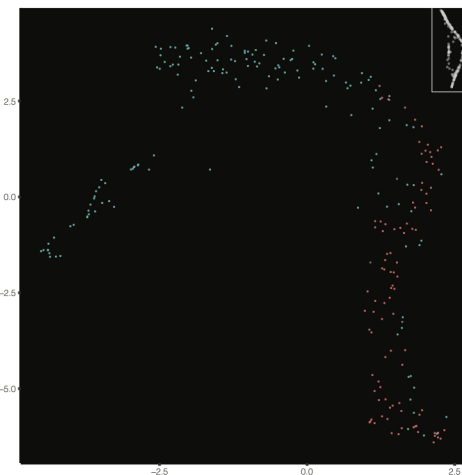

Cluster 4

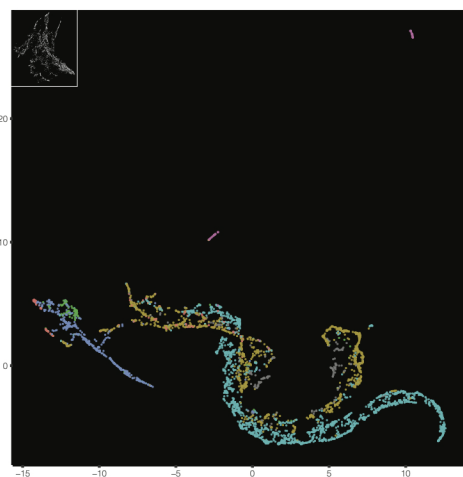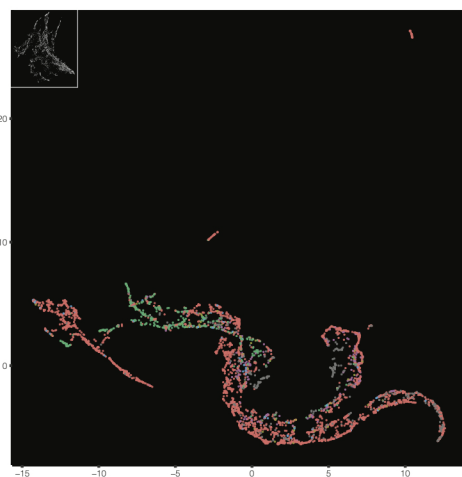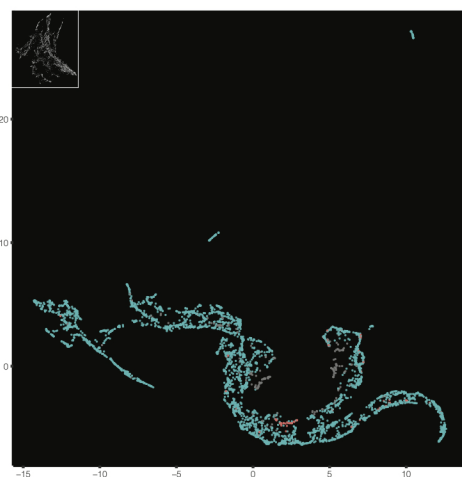

Cluster 7

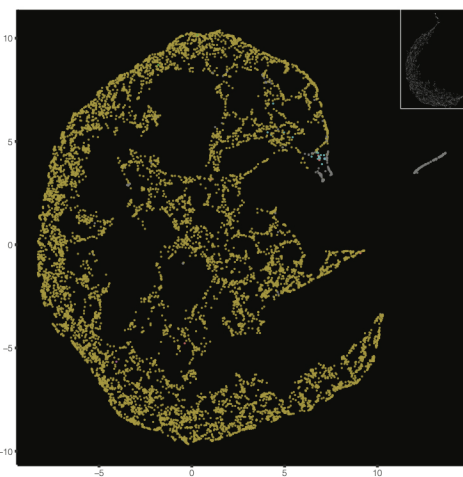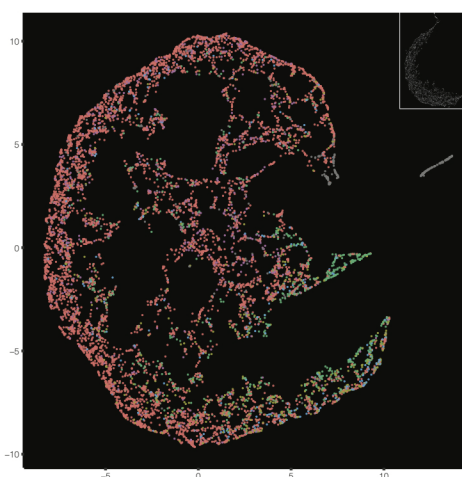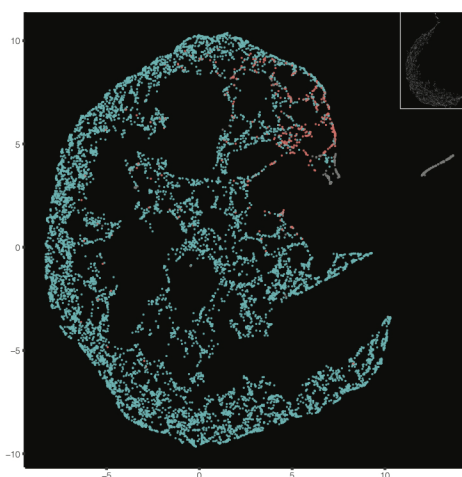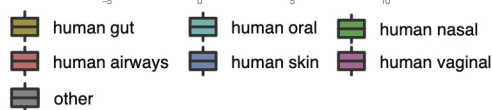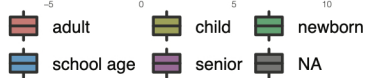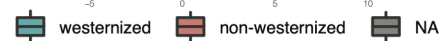

Supplement: FIG S6 [file msystems.00118-23-s0006.pdf]
