# Supplementary material for: Quantifying Shared and Unique Gene Content across 17 Microbial Ecosystems
Source: mSystems. 2023 Apr 6;8(2):e00118-23. doi: 10.1128/msystems.00118-23 (PMC10134805; doi:10.1128/msystems.00118-23)
Supplement: Reviewer comments [file reviewer-comments.pdf]

AMERICAN SOCIETY FOR MICROBIOLOGY | mSystems®

Quantifying shared and unique gene content across 17 microbial ecosystems

Samuel Zimmerman, Braden Tierney, Chirag Patel, and Aleksandar Kostic

Corresponding Author(s): Aleksandar Kostic, Joslin Diabetes Center

Review Timeline:

Submission Date:	February 8, 2023
Accepted:	February 27, 2023

Editor: Huilin Li

Reviewer(s): The reviewers have opted to remain anonymous.

Transaction Report:

(Note: With the exception of the correction of typographical or spelling errors that could be a source of ambiguity, letters and reports are not edited. The original formatting of letters and referee reports may not be reflected in this compilation.)

DOI: https://doi.org/10.1128/msystems.00118-23

February 27, 2023

Dr. Aleksandar D Kostic
Joslin Diabetes Center
Section on Pathophysiology and Molecular Pharmacology
One Joslin Place
Boston, Massachusetts 02215

Re: mSystems00118-23 (Quantifying shared and unique gene content across 17 microbial ecosystems)

Dear Dr. Aleksandar D Kostic:

Your manuscript has been accepted, and I am forwarding it to the ASM Journals Department for publication. For your reference, ASM Journals' address is given below. Before it can be scheduled for publication, your manuscript will be checked by the mSystems production staff to make sure that all elements meet the technical requirements for publication. They will contact you if anything needs to be revised before copyediting and production can begin. Otherwise, you will be notified when your proofs are ready to be viewed.

ASM policy requires that data be available to the public upon online posting of the article, so please verify all links to sequence records, if present, and make sure that each number retrieves the full record of the data. If a new accession number is not linked or a link is broken, provide production staff with the correct URL for the record. If the accession numbers for new data are not publicly accessible before the expected online posting of the article, publication of your article may be delayed; please contact the ASM production staff immediately with the expected release date.

As an open-access publication, mSystems receives no financial support from paid subscriptions and depends on authors' prompt payment of publication fees as soon as their articles are accepted.

Publication Fees: We have partnered with Copyright Clearance Center to collect author charges. You will soon receive a message from no-reply@copyright.com with further instructions. For questions related to paying charges through RightsLink, please contact Copyright Clearance Center by email at ASM_Support@copyright.com or toll free at +1.877.622.5543. Hours of operation: 24 hours per day, 7 days per week. Copyright Clearance Center makes every attempt to respond to all emails within 24 hours. For a complete list of **Publication Fees**, including supplemental material costs, please visit ourwebsite.

Corresponding authors may join or renew ASM membership to obtain discounts on publication fees. Need to upgrade your membership level? Please contact Customer Service at Service@asmusa.org.

If you would like to submit a potential Featured Image, please email a file and a short legend to msystems@asmusa.org. Please note that we can only consider images that (i) the authors created or own and (ii) have not been previously published. By submitting, you agree that the image can be used under the same terms as the published article. File requirements: square dimensions (4" x 4"), 300 dpi resolution, RGB colorspace, TIF file format.

For mSystems research articles, you are welcome to submit a short author video for your recently accepted paper. Videos are normally 1 minute long and are a great opportunity for junior authors to get greater exposure. Importantly, this video will not hold up the publication of your paper, and you can submit it at any time.

Details of the video are:

· Minimum resolution of 1280 x 720
· .mov or .mp4. video format
· Provide video in the highest quality possible, but do not exceed 1080p
· Provide a still/profile picture that is 640 (w) x 720 (h) max
· Provide the script that was used

We recognize that the video files can become quite large, and so to avoid quality loss ASM suggests sending the video file via https://www.wetransfer.com/. When you have a final version of the video and the still ready to share, please send it to mSystems staff at msystems@asmusa.org.

Thank you for submitting your paper to mSystems.

Sincerely,

Huilin Li
Editor, mSystems

Journals Department
American Society for Microbiology
1752 N St., NW
Washington, DC 20036
E-mail: mSystems@asmusa.org
